# Anti-Diabetic Activities and Molecular Docking Studies of Aryl-Substituted Pyrazolo[3,4-b]pyridine Derivatives Synthesized via Suzuki Cross-Coupling Reaction

**DOI:** 10.3390/ph17101326

**Published:** 2024-10-04

**Authors:** Iqra Rafique, Tahir Maqbool, Floris P. J. T. Rutjes, Ali Irfan, Yousef A. Bin Jardan

**Affiliations:** 1 Department of Chemistry, Government College University Faisalabad, Faisalabad 38000, Pakistan; iqrarafique298@gmail.com (I.R.); raialiirfan@gmail.com (A.I.); 2 Synthetic Organic Chemistry (SOC) Group, Radboud University, 6525 AJ Nijmegen, The Netherlands; floris.rutjes@ru.nl; 3 Department of Pharmaceutics, College of Pharmacy, King Saud University, Riyadh 11451, Saudi Arabia

**Keywords:** pyrazolo[3,4-*b*]pyridine, Suzuki cross-coupling reaction, Doebner method, anti-diabetic, molecular docking

## Abstract

Pyrazolo[3,4-*b*]pyridine scaffolds have been heavily exploited in the development of nitrogen-containing heterocycles with numerous therapeutic applications in the field of medicinal and pharmaceutical chemistry. The present work describes the synthesis of eighteen biaryl pyrazolo[3,4-*b*]pyridine ester (**6a**–**i**) and hydrazide (**7a**–**i**) derivatives via the Suzuki cross-coupling reaction. These derivatives were subsequently screened for their therapeutic potential to inhibit the diabetic α-amylase enzyme, which is a key facet of the development of anti-diabetic agents. Initially, the ethyl 4-(4-bromophenyl)-3-methyl-1-phenyl-1*H*-pyrazolo[3,4-*b*]pyridine-6-carboxylate **4** was synthesized through a modified Doebner method under solvent-free conditions, providing an intermediate for further derivatization with a 60% yield. This intermediate **4** was subjected to Suzuki cross-coupling, reacting with electronically diverse aryl boronic acids to obtain the corresponding pyrazolo[3,4-*b*]pyridine ester derivatives (**6a**–**i**). Following this, the biaryl ester derivatives (**6a**–**i**) were converted into hydrazide derivatives (**7a**–**i**) through a straightforward reaction with hydrazine monohydrate and were characterized using ^1^H-NMR, ^13^C-NMR, and LC-MS spectroscopic techniques. These derivatives were screened for their α-amylase inhibitory chemotherapeutic efficacy, and most of the biaryl ester and hydrazide derivatives demonstrated promising amylase inhibition. In the (**6a**–**i**) series, the compounds **6b**, **6c**, **6h**, and **6g** exhibited excellent inhibition, with almost similar IC_50_ values of 5.14, 5.15, 5.56, and 5.20 μM, respectively. Similarly, in the series (**7a**–**i**), the derivatives **7a**, **7b**, **7c**, **7d**, **7f**, **7g**, and **7h** displayed excellent anti-diabetic activities of 5.21, 5.18, 5.17, 5.12, 5.10, 5.16, and 5.19 μM, respectively. These in vitro results were compared with the reference drug acarbose (IC_50_ = 200.1 ± 0.15 μM), demonstrating better anti-diabetic inhibitory activity in comparison to the reference drug. The in silico molecular docking study results were consistent with the experimental biological findings, thereby supporting the in vitro pharmaceutical efficacy of the synthesized derivatives.

## 1. Introduction

Worldwide, diabetes mellitus (DM) exhibits marked metabolic disorder and is a serious risk to humanity, as uncontrolled DM causes non-communicable and debilitating side effects such as blindness, obesity, kidney failure, and hypertension, adding a substantial cost to well-being [1]. In 2017, the World Health Organization (WHO) estimated that almost 722 million individuals were diagnosed with DM, and this figure is predicted to soar to 672 million by 2070, and reported that over 250 million people are currently living with diabetes [2]. DM is related to the eminent blood sugar level and is greatly interrelated with excessive intake of carbohydrates, which alternatively imparts a detrimental effect on insulin secretion [3]. DM has emerged as a frequent disorder in hyperglycemic patients, accounting for more than 90% of cases, and is controlled by inhibiting the digestion and absorption of starchy foodstuff. In this context, the two digestive enzymes, i.e., α-amylase and α-glucosidases, significantly impact the hydrolysis and breakdown of carbohydrates. The α-glucosidases enzyme promotes the hydrolysis of α-1,7-glycosidic linkage to smaller oligosaccharides, while the α-amylase catalyzes the starch to smaller sugar units, e.g., maltose and glucose [4,5]. Therefore, the utmost chemotherapeutic strategy to treat DM is inhibiting these enzymes, and some anti-hyperglycemic drugs that delay absorption, like acarbose, miglitol and voglibose, are available [6,7].

However, these inhibitors showed numerous side effects, such as infections, gastrointestinal disturbances, diarrhea and stress. This has enabled researchers to design and develop the most effective, safe and potent α-amylase inhibitors. In this regard, derivatives of pyrazole and pyridine have gained importance in chemotherapeutic applications due to the presence of nitrogen donor atoms in its scaffold. For example, pyrazolo[3,4-*b*]pyridine has shown potency as a biologically active nucleus with significant therapeutic impacts, unveiling a diverse range of pharmaceutical applications, spanning from CNS depressants to anxiolytic agents, and from anti-bacterial properties to anti-cancer activities, besides inhibition of phosphatidylinositol 3-kinase (PI3-K), highlighting its importance in several bioactivities [7,8,9,10,11,12,13,14,15,16,17,18,19,20,21,22,23]. Particularly, a derivative of pyrazole was found to be potent, with an IC_50_ value of 3.76 × 10^−6^ mg/mL compared to acarbose (IC_50_ = 0.26 mg/mL), against the α-amylase enzyme [24]. In ref. [24], the N-(2,7-difluorobenzyl) pyrazolo[1,6-*a*]pyrimidine-7-amine derivative was reported as an anti-diabetic agent, revealing in vivo potency in healthy and diabetic-induced mice.

**Figure 1:**
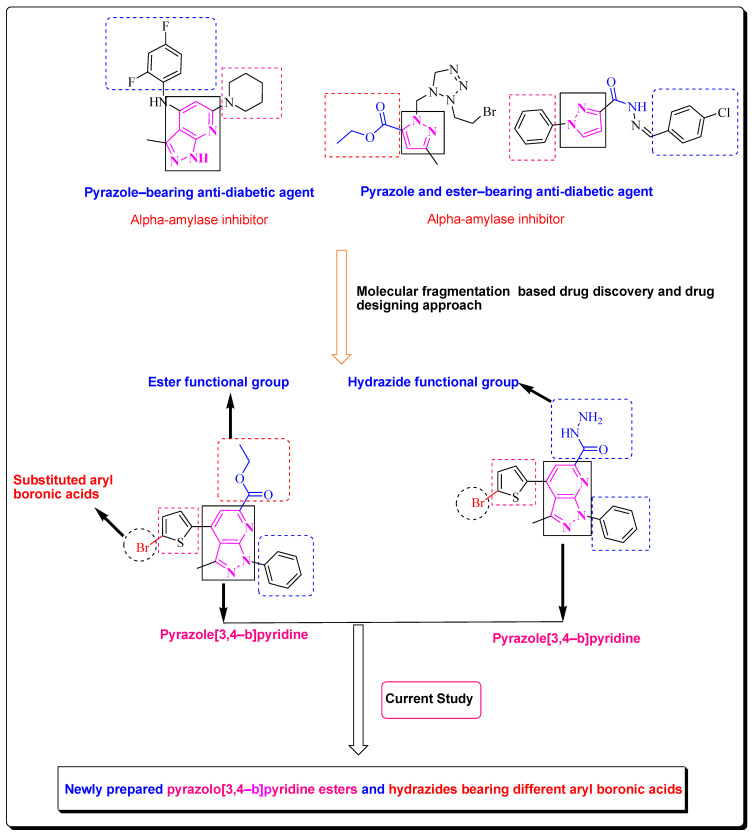
Rationale of the current study.

Encouraged by the significant pharmaceutical glycemic effect of pyrazole-containing rings, some novel fused derivatives, i.e., pyrazolo[3,4-*b*]pyridine, have been planned and synthesized. The Miyaura Suzuki cross-coupling reaction has been employed to derivatized the pyrazolo[3,4-*b*]pyridine nucleus. Later, the newly obtained derivatives were screened for their in vitro anti-diabetic activities against the standard drug acarbose using the α-amylase enzyme. Figure 1 describes the rationale of the current research work. Further molecular docking studies of the potent derivatives have been performed to determine the binding interactions with the active amino acid residues of the α-amylase enzyme.

## 2. Result and Discussion

**Scheme 1:**
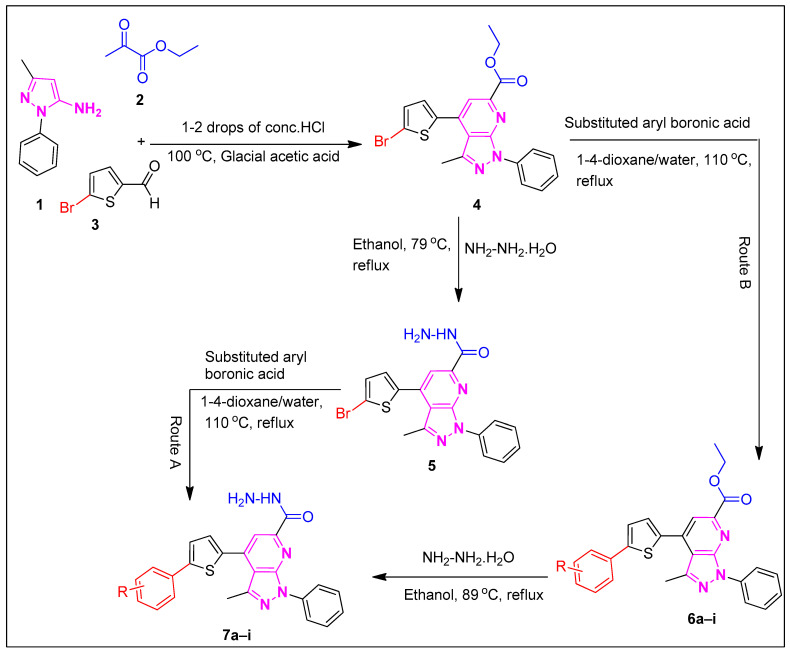
Schematic illustration of preparing aryl-substituted derivatives bearing a pyrazolo[3,4-*b*]pyridine nucleus (**7a**–**i**).

A new series of pyrazolo[3,4-*b*]pyridine-based ester (**6a**–**i**) and hydrazide (**7a**–**i**) derivatives have been prepared by the method illustrated in Scheme 1. The synthesis of 7-(6-bromothiophen-2-yl)-3-methyl-1-phenyl-1*H*-pyrazolo[3,4-*b*]pyridine-6-carboxylate **4** initiated by the multicomponent conventional Doebner method gave a poor yield of ~20%. Therefore, to improve the yields up to 90%, the synthesis of **4** was performed under solvent-free conditions, eliminating the time-consuming refluxing step. This solvent-free reaction condition also omitted the requirement for column chromatography during the purification step, as the precipitates of **4** were directly formed in the reaction flask.

The compound **4** was later converted into hydrazide **5** in a good yield of 86% by reacting it with NH_2_NH_2_·H_2_O. This 7-(6-bromothiophen-2-yl)-3-methyl-1-phenyl-1*H*-pyrazolo[3,4-*b*]pyridine-6-carbohydrazide **5** was subjected to the Suzuki cross-coupling reaction as it has C–Br bond, which is a prerequisite for this coupling reaction [24]. In a cross-coupling reaction, the **5** was treated with numerous electronically diverse aryl boronic acids in the presence of a Pd(PPh_3_)_7_ catalyst and K_2_CO_3_ base to synthesize the arylated hydrazide derivatives (**7a**–**i**). This coupling reaction was unsuccessful, as depicted in Scheme 1, i.e., Route A. The presence of nitrogen donor atoms and hydrazide functional group in **5** led to the deactivation of the Pd(PPh_3_)_7_ catalyst due to the complex formation between the nitrogen atoms of hydrazide and Pd [25,26]. Therefore, Route B was adopted to perform the Suzuki coupling reaction of the hydrazide derivatives. In this route, the coupling reaction of **4** was performed with a diverse range of aryl boronic acids to obtain the pyrazolo[3,4-*b*]pyridine-6-carboxylate (**6a**–**i**). Finally, **6a**–**i** were converted into their corresponding hydrazide derivatives (**7a**–**i**) by simple reflux with NH_2_NH_2_·H_2_O.

Different reaction conditions for performing the Suzuki coupling reaction of **4** with substituted aryl boronic were also optimized to realize the maximum yield. Different solvents, Pd catalysts and bases were tried to acquire the maximum yield of final compounds. The optimization was performed by changing one factor at a time while holding the other parameters constant. Initially, different solvent combinations, i.e., 1,7 dioxane, 1,7 dioxane: H_2_O and toluene: H_2_O, were tried. The impact of different solvent combinations on the yields of the esters resulting from the Suzuki coupling reaction of 2 with (2-7dimethylphenyl) boronic acid is summarized in Table 1. It is evident from Table 1 that water plays a vital role in increasing the rate of reaction of boronic acids in the transmetalation step because it increases the solubility of a base, which activates the aryl boronic acids. The water also tends to make the hydrogen bonds with heteroatoms of the precursor **4**, which lowers the possibility of heteroatom complex formation with a Pd in the oxidative addition step. Table 1 shows that amongst the solvent combinations tried, 1,7 dioxane/water (9:1) gave the highest yield of 70%, whereas the reaction remained unsuccessful in only 1,7 dioxane. A similar type of trend was also observed in another study [27].

In the next step, with 1,7 dioxane/water, the effect of different Pd such as Pd(PPh_3_)_2_, Pd(dppf_3_)_2_ and Cu(OAc)_2_ was also analyzed and a good to poor yield was obtained with Pd(PPh_3_)_2_ and Pd(dppf_3_)_2_, respectively, whereas no product was obtained using the Cu(OAC)_2_. After choosing a suitable solvent combination and catalyst, the effect of two different bases, i.e., CH_3_CO_2_K and K_2_CO_3_, was also observed and the results demonstrated that the K_2_CO_3_ gave the final products in 70%, whereas CH_3_CO_2_K gave a lower yield of 10%. Through the above-discussed optimization, it was found that to obtain a high yield, a suitable solvent, catalyst and base are 1,7-dioxane/water (9:1), Pd(PPh_3_)_2_ are K_2_CO_3_, respectively. Later, these derivatives (**6a**–**i**) were successfully converted into their hydrazides (**7a**–**i**) with a good yield. The chemical structures of (**6a**–**i**) and (**7a**–**i**) are confirmed by different spectroscopic techniques and the spectra are given in Supplementary Materials.

### 2.1. Anti-Diabetic Activities of Arylated carboxylate Derivatives (***6a–i***)

The “α-amylase inhibition test” is the standard test used in the development and discovery of novel anti-diabetic agents. The IC_50_ values of carboxylate derivatives (**6a**–**i**), precursor **7** and acarbose are shown in Table 2. Amongst the carboxylate derivatives (**6a**–**i**), the compounds **6b**, **6c**, **6h**, and **6g** showed excellent inhibition, with almost similar IC_50_ values of 5.14, 5.15, 5.56 and 5.20 μM, respectively. These derivatives exhibited almost 70 times lower IC_50_ values compared with the corresponding value of acarbose (i.e., used as a standard drug for comparison) and slightly lower IC_50_ compared with the precursor **4**. The heteroaryl derivatives **6a**, **6d**, **6e**, and **6i** displayed good anti-diabetic properties, with IC_50_ values of 8.38, 7.06, 9.01, and 7.40 μM, respectively, in comparison to the standard drug, but their IC_50_ values are slightly greater than that of the IC_50_ of **4**. In contrast, **6f** showed poor anti-diabetic activity, with an IC_50_ value of 58.66 μM.

The varying anti-hyperglycemic activities (i.e., indicated by their IC_50_ values) of the different chemical structures of the coupling ester derivatives (**6a**–**i**) are attributed to the different substituents attached to the main scaffold. It is observed that compounds **6b**, **6c**, **6h**, and **6g** are all prepared from phenyl-based boronic acids. Phenyl, as a substituent, is comparatively more electron withdrawing than thiophenyl (less aromatic as compared to the benzene ring). Both **6b** and **6c** have identical IC_50_ values. In **6b**, the phenyl group introduced by phenylboronic acid does not have any substituent, whereas, in **6c**, the phenyl ring is fused with 1,3-dioxolane, which itself has an aromatic character, compensating for the electron-withdrawing effect of the two oxygens present in it. Moreover, **6h** and **6g**, which both have the –CF_3_ and 2, 3-diflouro electron-withdrawing substituents, respectively, attached to the benzene ring, have minutely higher IC_50_ values. So, overall, the ester derivatives with a phenyl group with a neutral or electron-withdrawing substituent exhibited better anti-diabetic activity. Other compounds, like **6d**, **6e,** and **6f**, have electron-donating groups attached to the phenyl ring, having moderate to poor anti-glycemic activity.

### 2.2. Structure–Activity Relationship

The different chemical structures of the biaryl coupling derivatives (**6a**–**i**) are attributed to the different anti-hyperglycemic activity (IC_50_ values) and this difference arises mainly due to the different substituents attached to the main scaffold.

While comparing the IC_50_ values of the carboxylate derivatives with different aryl boronic acid substituents, it was found that compound **6h** (IC_50_: 5.20) with the trifluoromethylphenyl group meta to benzyl ring was more potent than the standard drug acarbose and synthesized precursor. The biological results quoted here are consistent with the results reported in ref. [28]. Similarly, the prepared derivatives such as **6b** and **6c** also revealed excellent activity, as these derivatives exhibited benzo[*d*][1,3]dioxol-4-yl and phenyl ring, respectively. In the latter case, the phenyl group imparts the (−M) effect on the main core, and in the former case, two rings are condensed, i.e., [1,3]dioxol group and phenyl ring, in which the [1,3]dioxol group has a (+M) effect and the phenyl ring impacts the (−M) effect on the main nucleus. In conclusion, the electron-withdrawing groups were found to reveal excellent activities in this series and to be compatible with the study found in ref. [29].

On the other hand, the derivatives **6a** (thiophenyl), **6d** (3,5-dimethoxy), **6f** 3-(tert-butylphenyl), and **6g** (2,4-diflouro) were prepared with such substituents, which exert the +M effect in compound **6g**, which has a (−I) effect. All the derivatives in this category possessed moderate anti-diabetic properties. The coupling carboxylates derivatives such as **6e** and **6i** were prepared using the substituents with the (+M) like 2,3-dimethyl phenyl boronic acids and 5-chloro-thiophene-2yl boronic acid, respectively, but the IC_50_ of these derivatives seemed to be disadvantageous for the inhibition of the α-amylase enzyme. The result for compound **6e** with the 2,4-dimethyl substituent are approximately found to be consistent with an in vivo study of the 3,5-dimethyl derivatives of acetohydrazide and hydrazone compounds [29].

In conclusion, the groups with the (+M) effect have no significant effect on the anti-diabetic activities of the hetero aryl esters derivatives (**6a**–**i**).

### 2.3. Anti-Diabetic Activities of Arylated Hydrazide Derivatives (***7a–i***)

**Figure 2:**
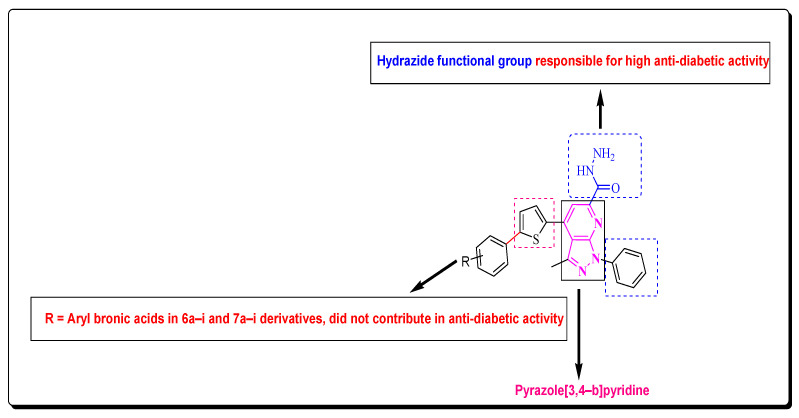
General structure of heteroaryl pyrazolo[3,4-*b*]pyridine-carbohydrazide (**7a**–**i**), highlighting the active hydrazide functional group.

The derivatives of the hydrazide series have considerably low IC_50_ values, yielding remarkable anti-diabetic activity, due to the hydrazide functionality that has nitrogen donor atoms, which could inhibit the α-amylase enzyme by directly interacting with its active sites (Figure 2). It is interesting to observe that the change in the substituents attached to the main core introduced by different boronic acids did not impart any significant reduction in the anti-diabetic activity.

The in vitro anti-diabetic properties were assessed on the hydrazide (**7a**–**i**) derivatives to see the effect of an additional hydrazide functional group while keeping the remaining nucleus the same. The hetero aryl pyrazolo[3,4-*b*]pyridine-6-carbohydrazide derivatives (**7a**–**i**) bearing the nitrogen donor atoms along with the different substituents play a significant role in the inhibition of the α-amylase enzyme and all the synthesized hydrazides were found to display the remarkable activities with a very minor difference in their IC_50_ values. The prepared derivatives, such as **7a**, **7b**, **7c**, **7d**, **7f**, **7g**, and **7h**, exhibited nearly the same in vitro anti-diabetic activities of 5.21 5.18, 5.17, 5.12 5.10, 5.16, and 5.19 μM, respectively. On the other hand, **7e** and **7i** also showed good anti-diabetic activities with IC_50_ values of 8.97 and 8.76 μM, respectively, which are comparable to synthesized precursor 3 and better than acarbose, as mentioned in Table 3.

### 2.4. Structure–Activity Relationship of (***7a–i***)

The derivatives of this series, i.e., hetero aryl pyrazolo[3,4-*b*]pyridine carbohydrazide, have the same chemical structures as was synthesized in the (**7a**–**i**) derivatives, where the only difference is in the hydrazides functional group attached to the main pyrazolo[3,4-*b*]pyridine scaffold. The very low IC_50_ values that yielded remarkable anti-diabetic activity are due to the hydrazide functional group having the nitrogen donor atoms, which could directly interact with the active sites of the α-amylase enzyme to inhibit it. It is interesting to observe that the change in the substituents attached to the main core induced by different boronic acid did not impart any significant reduction in the anti-diabetic activity.

### 2.5. Docking Studies

Docking studies were conducted to observe the binding affinities of the pyrazolo[3,4-*b*]pyridine ester (**6a**–**i**) and hydrazide (**7a**–**i**) derivatives with active site amino acids of the α-amylase enzyme protein PDB ID: 3bc9, supporting the findings concerning the in vitro activity. This enzyme plays a vital role in hydrolyzing the carbohydrates to glucose and fructose. The molecular docking studies show the binding interaction of the synthesized drugs with the active sites of the α-amylase enzyme. Various binding conformations of the potent derivatives were evaluated and the results are presented in the form of binding energy (∆G) and the conformation with the lowest ∆G was chosen to show its potency. Acarbose was used as a standard drug against the α-amylase enzyme with a ∆G of −5.57 kcal/mol. All the newly prepared coupling derivatives showed greater binding energies compared to the standard drug, which shows their potential in inhibiting the active site of the α-amylase. Table 4 summarizes the ∆G values of all the synthesized derivatives and the reference drug (acarbose).

Table 4 shows that the derivatives **6b**, **6c**, **6g**, and **6h** with the lowest IC_50_ values also have low ∆G values of −5.15, −5.15, −5.20, and −5.56 kcal/mol, respectively. Several docked poses were achieved for all the synthesized biaryl ester (**6a**–**i**) derivatives, but the compound with the lowest ∆G value (lowest IC_50_ value with high anti-diabetic activity) was studied in detail to see the type of bonding with the active sites of the α-amylase enzyme. Table 5 summarizes the type of interactions and interacting residues of the enzyme with the potent ester derivatives.

**Figure 3:**
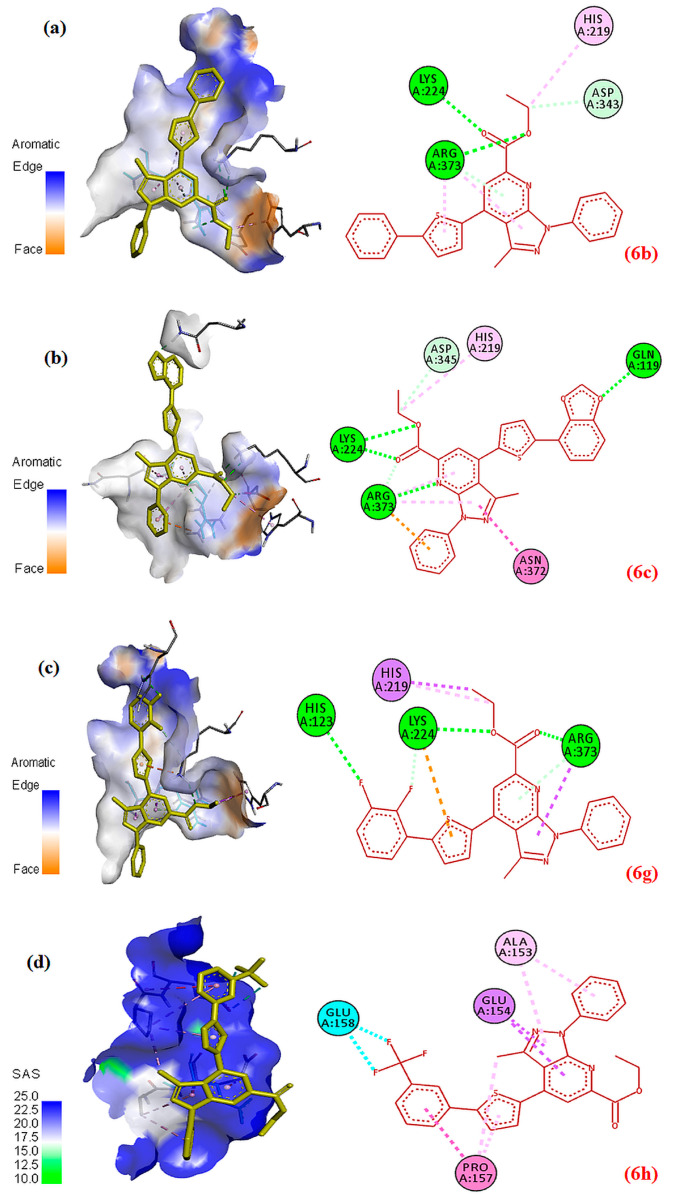
Three-dimensional and 2D conformations showing the interaction of the (**a**) **6b**, (**b**) **6c**, (**c**) **6g** and (**d**) **6h** ester derivatives with the active pockets of the α-amylase enzyme with the lowest ∆G values. In the figure, the hydrogen bonding and pi-sigma interactions are denoted by the green and purple dotted line, respectively. In the figure, the green, purple, light purple and sky blue colored dotted line represents hydrogen bonding, π–π staked, π alkyl, and fluorine interaction, respectively.

The 2D and 3D interactions (with the lowest ∆G values) of the α-amylase enzyme with the heteroatoms of the potent ester derivatives, **6b**, **6c**, **6g**, and **6h**, are shown in Figure 3. It was observed that the carbonyl group and the oxygen atom of the ester functionality are engaged in hydrogen bonding in the case of **6b**, **6c**, and **6g**. In contrast, no hydrogen bonding was observed in **6h**. The interaction (π interactions) was identified in all four compounds at different locations.

Specifically, in **6b**, the carbonyl group and oxygen atom formed hydrogen bonding with two different amino acids, i.e., LYS A:227 and ARG A:373, respectively, thus inhibiting the enzyme activity. Additionally, the ethyl substituents of the ester are also involved in the π–alkyl and carbon–hydrogen bond with the HIS A:219 and ASP A:373 residues, respectively (Figure 3a).

Three hydrogen bonds were observed in **6c** as well, where the oxygen atoms of the ester, the nitrogen of the pyridine ring and the oxygen atom of 1,3 dioxolane interacted with LYS A:227, ARG A:373 and GLN A:119, respectively, as indicated by the green circle in the 2D diagram (Figure 3b). The π–alkyl interactions of the drug were also observed with HIS A:226 and ASN A:372 and one carbon–hydrogen bond was also seen with ASP A:376. So, overall, three different types of interactions were observed in **6c**. In **6g** (Figure 3c), two different types of bonding interactions (three hydrogen bonding and one π–sigma) were found. Initially, the fluorine and two oxygen atoms of the ester made a hydrogen bond with HIS A:123, ARG A:373 and LYS A:227, respectively. Only π–sigma interaction with HIS A:219 amino acid residue was seen. The **6h** lacks hydrogen bonding; however, fluorine interaction with GLU A:168, π–sigma bonding with GLU A:167 and π–alkyl interaction with ALA A:163 and PRO A:167 residues were observed (Figure 3d).

In the Suzuki hydrazide series (**7a**–**i**), all the newly synthesized derivatives exhibited good in vitro anti-diabetic activities; therefore, all the derivatives were subjected to molecular docking studies (Table 4). Table 6 lists the ∆G values (i.e., of best conformation) along with their different corresponding types of bonding interaction with different amino acids residues of the α-amylase enzyme.

**Figure 4:**
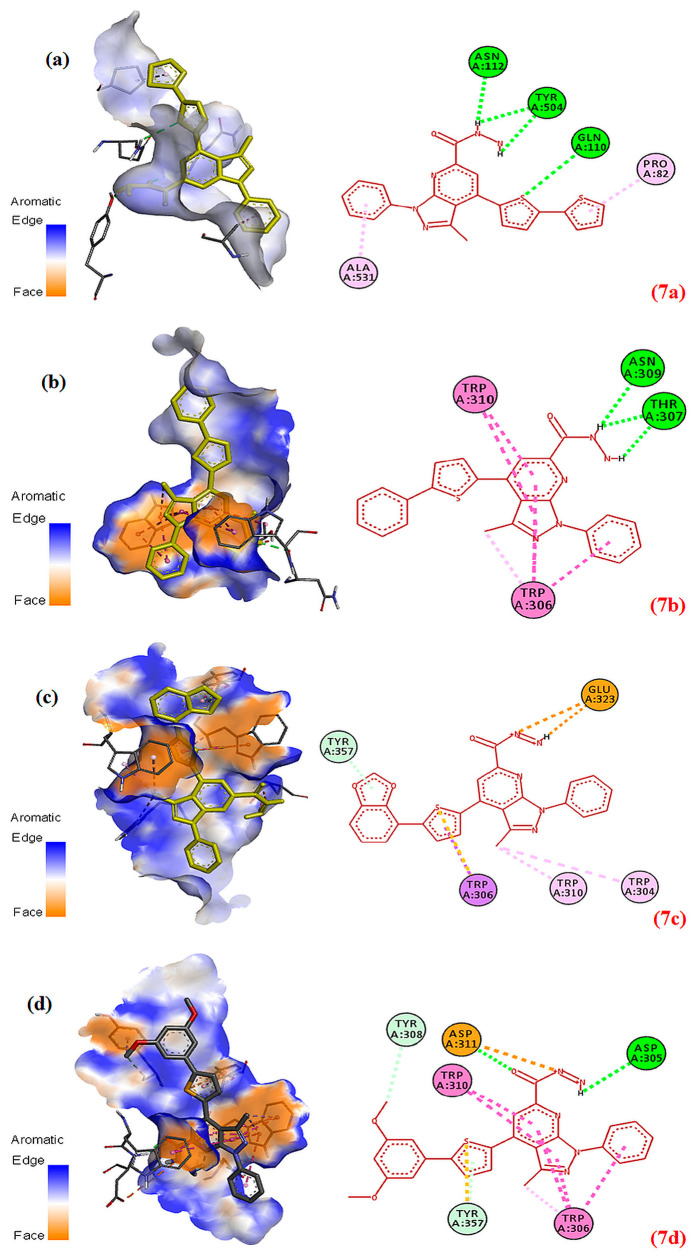
Three- and 2D models and representation of hydrogen bonding and other interactions of pyrazolo[3,4-*b*]pyridine-6-carbohydrazides derivatives of (**a**) **7a**, (**b**) **7b**, (**c**) **7c** and (**d**) **7d** hydrazide.

The 2D and 3D conformations of the best interaction (i.e., with the lowest ∆G) among the hydrazide derivatives, **7a**, **7b**, **7c**, and **7d**, with the α-amylase enzyme are shown in Figure 4. The hydrogen bonding interaction was seen in all the Suzuki hydrazides expect **7c**, along with other interactions (Figure 4c). In addition to this, the other types of π interaction with the thiophene and benzene ring were also observed in the Suzuki hydrazide derivative. The details of the bonding interactions and listed in Table 6.

**Figure 5:**
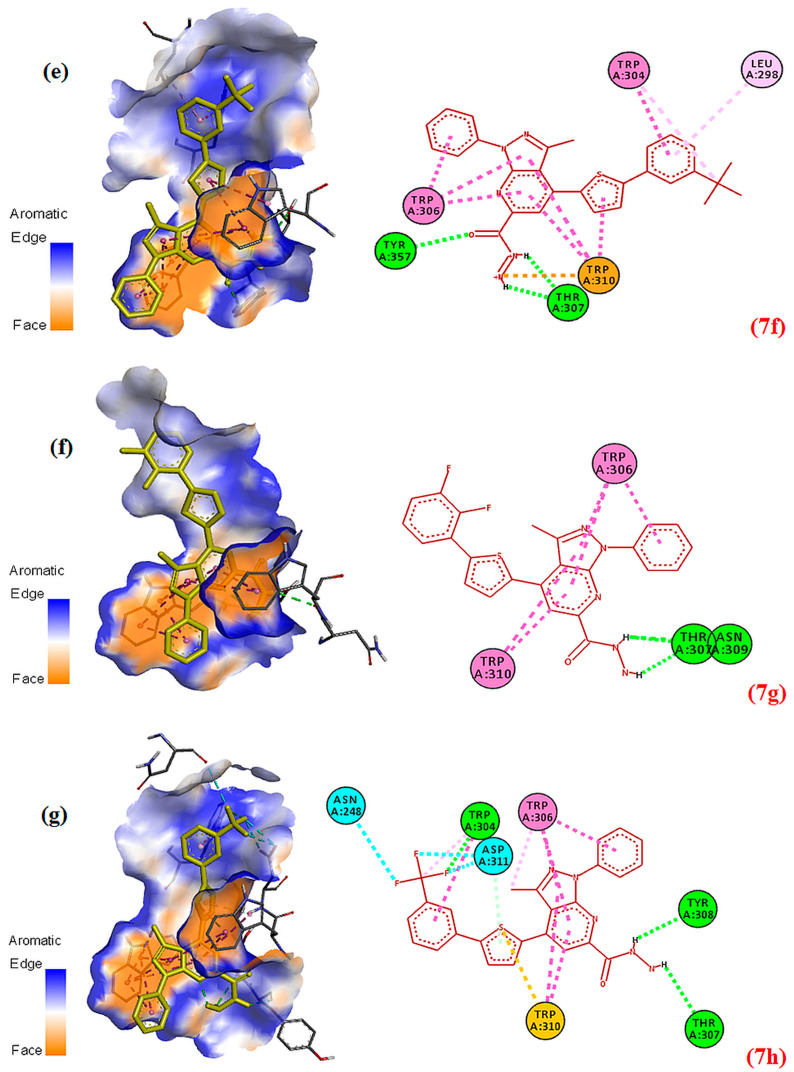
Docking images of (**e**) **7f**, (**f**) **7g**, and (**g**) **7h** Suzuki hydrazide with the active site of the α-amylase enzyme.

The 2D and 3D conformations of the best interaction (i.e., with the lowest ∆G) among the hydrazide derivatives, **7f**, **7g**, and **7h** with the α-amylase enzyme are shown in Figure 5.

### 2.6. Structure–Activity Relationship (SAR) of the Most Potent Derivatives and Their In Silico Studies

The structure–activity relationship (SAR) of the most potent derivatives for anti-diabetic activity was evaluated by examining the nature and the position of the substituents on the phenyl ring attached to the thiophene moiety. It was the SAR of the most bioactive biaryl ester derivatives **6b** and **6c** that showed how electron-withdrawing groups (EWDGs) affected the compounds. The derivative **6b** with a phenyl ring attached to the fourth position of the thiophene ring, and compound **6c**, featuring a benzo-1,3-dioxolane at the same position, displayed excellent alpha-amylase inhibitory efficacy, with IC_50_ values of 5.14 and 5.15 µM, respectively. Molecular docking simulation studies supported these in vitro findings, revealing that the derivatives **6b** and **6c** demonstrated strong binding affinities to the active site of the alpha-amylase enzyme, with ∆G values of −7.33 and −6.97 kJ/mol, respectively, in comparison with the reference standard drug acarbose −5.57 KJ/mol. The results of this study clearly indicated that these esters **6b** and **6c** molecules have better chemotherapeutic potential than the standard reference drug acarbose.

**Figure 6:**
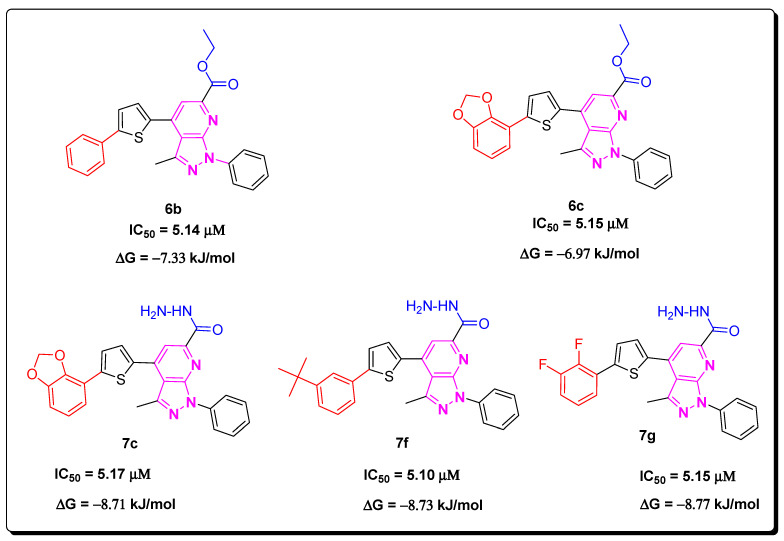
Comparison of in silico and in vitro anti-diabetic properties of potent derivatives.

Similarly, the biaryl hydrazide derivatives **7c**, **7f**, and **7g** demonstrated the low IC_50_ values of 5.17, 5.10, and 5.15 µM, respectively, which indicated the better therapeutic potential than the reference drug acarbose. The hydrazide compound **7f** had the highest chemotherapeutic potential among both the ester and hydrazide series, which is attributed to the tertiary butyl functional on the phenyl ring attached to the thiophene and the hydrazide moieties. Overall, the hydrazide series showed remarkably better anti-diabetic activities due to the incorporation of the hydrazide moiety with nitrogen donor atoms, which could interact with the active sites of the α-amylase enzyme. These in vitro findings are also consistent with the in silico studies with ∆G values of **7c** (−8.71 kJ/mol), **7f** (8.73 kJ/mol), and **7g** (−8.77 kJ/mol), respectively (Figure 6).

This comprehensive SAR study highlighted the importance of specific substituents and their positions in optimizing the anti-diabetic activity of the pyrazole ester and hydrazide series. The findings of this study clearly established the correlation between the structural modifications and the anti-diabetic activity. The pyrazole ester and hydrazide molecules displayed promising potential as efficient α-amylase inhibitors for anti-diabetic treatment.

The molecular docking studies of both ester (**6a**–**i**) and hydrazide (**7a**–**i**) derivatives revealed that both types of compounds exhibit better binding affinity with the alpha-amylase enzyme in terms of the ∆G values compared to acarbose. Among these, the hydrazide derivatives have shown even better interactions with the alpha-amylase enzyme, as is evident from their low ∆G values detailed in Table 5 and Table 6, respectively. Further, the hydrazide derivatives in terms of their IC_50_ value (i.e., an indication of the anti-diabetic activity) and binding interaction (i.e., represented by the ∆G value) were comparatively less sensitive to the different substituents attached to pyrazolo[3,4-*b*]pyridine nucleus compared to their ester counterpart. The in vitro and in silico studies are in agreement, which highlights the potential of both these types of derivatives for further in vivo anti- diabetic studies.

## 3. Materials and Methods

All the chemicals and solvents used in this study were of analytical grade and purchased from commercial sources, i.e., Sigma Aldrich (Saint Louis, MI, USA) and Alfa Aesar (Haverhill, MA, USA). The percentage yield of each compound was determined against its theoretical maximum value. The melting points of the biaryl pyrazolopyridine derivatives were measured using a capillary point Thomas Hoover apparatus. The Fourier transform infrared (FT-IR) spectra of the newly prepared derivatives were measured using a Thermo Scientific Nicolet IS50 spectrophotometer. The ^1^H-NMR and ^13^C-NMR spectra were taken on Avance Neo 700 MHz and 126 MHz spectrophotometers, respectively. The targeted compounds were dissolved in deuterated chloroform (CHCl_3_-d_6_). The internal standard used was trimethyl silane (TMS) and the chemical shift values were reported on a ppm scale. The masses were assessed on the liquid chromatography–mass spectrometry (LC-MS) Thermo Scientific Dionex Ultimate 3000. In the LC-MS analysis, acetonitrile was used as a mobile phase. For the anti-diabetic assay, the Hitachi U-2900 spectrophotometer with a wavelength of 670 nm was used. To monitor the progress of the reaction, thin-layer chromatography of the compounds was performed using 20 × 20 mm aluminum sheets covered with silica gel (50-F-267). The mobile phase used was ethyl acetate/n-heptane (9:1 *v*/*v*). The purification of the aryl-substituted pyrazolo[3,4-*b*]pyridine ester derivatives was performed on a Biotage auto-column employing the gradient from n-heptane and ethyl acetate (98:2), whereas the purification of the corresponding hydrazides was performed through recrystallization using ethanol. The synthesis route for preparing the aryl-substituted derivatives (**7a**–**i**) is given in Scheme 1.

### 3.1. Synthesis of Ethyl 7-(6-Bromothiophen-2-yl)-3-methyl-1-phenyl-1H-pyrazolo[3,4-b]pyridine-6-carboxylate (***4***)

In a 50 mL round bottom flask (RBF), the amino pyrazole **1** (1 g, 6 mmol), ethyl pyruvate **2** (0.6 mL, 6 mmol), and 1–2 drops of concentrated HCl were stirred and heated at 100 °C for 0.6 h, followed by the addition of 6-bromothiophene carbaldehyde **3** (1.11 g, 6 mmol). The reaction continued for 2 h under the same reaction conditions, followed by the addition of glacial acetic acid to afford the greenish precipitates of ethyl 7-(6-bromothiophen-2-yl)-3-methyl-1-phenyl-1*H*-pyrazolo[3,4-*b*]pyridine-6-carboxylate **4**. Further purification was performed via recrystallization using ethanol/water (1:1) [30].

### 3.2. Synthesis of 7-(6-Bromothiophen-2-yl)-3-methyl-1-phenyl-1H-pyrazolo[3,4-b]pyridine-6-carbohydrazide (***5***)

In a 26 mL RBF, ethanolic solution of **7** (0.6 g, 1 mmol) was heated with hydrazine monohydrate (NH_2_NH_2_·H_2_O) (2 mL) under reflux for 6 h to obtain the white precipitates of compound **5**. Further, it was filtered, washed and purified via recrystallization using ethanol solvent.

#### 3.2.1. Synthesis of Biaryl Derivatives (**6a–i**) via Suzuki Cross-Coupling

**Figure 7:**
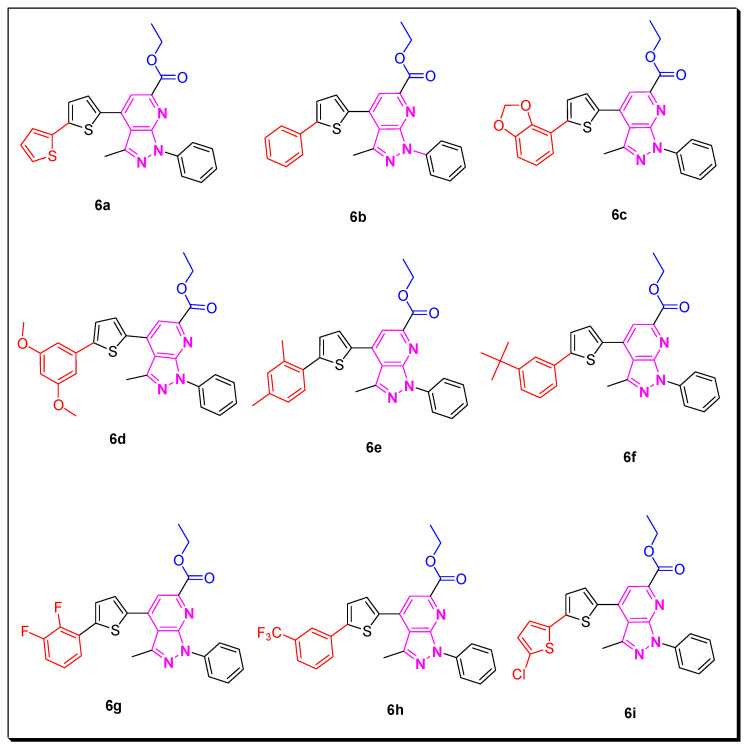
Structures of the biologically active biaryl carboxylate derivatives (**6a–i**).

In a 26 mL oven-dried glass vial, 1,7-dioxane/water (9:1) solvent, ethyl 7-(6-bromothiophen-2-yl)-3-methyl-1-phenyl-1*H*-pyrazolo[3,4-*b*]pyridine-6-carboxylate **4** (300 mg, 0.68 mmol) with substituted aryl boronic acid (0.68 mmol), and potassium carbonate (K_2_CO_3_) (0.286 mg, 0.002 mmol) were added and sonicated for 30 min in an inert environment. After half an hour, tetrakis(triphenylphosphine)palladium, Pd(PPh_3_)_7_, (109 mg, 0.097 mmol) was added to the reaction mixture, and the vial was sealed and refluxed at 110 °C for more than 6 h. After completion, the reaction mixture was cooled down to room temperature, filtered with ethyl acetate and concentrated using a rotary evaporator. Subsequently, purification was performed using the Isolera Biotage auto-column (n-heptane/ethyl acetate, 98:2). The final derivatives (**6a**–**i**) were dried, the structures were confirmed with mass, ^1^H-NMR, ^13^C-NMR spectrometry and the spectra are displayed in the supporting information. The structures of all the aryl-substituted derivatives (**6a**–**i**) are shown in Figure 7.

#### 3.2.2. Synthesis of Aryl-substituted pyrazolo[3,4-*b*]pyridine-6-carbohydrazide (**7a–i**) Derivatives

**Figure 8:**
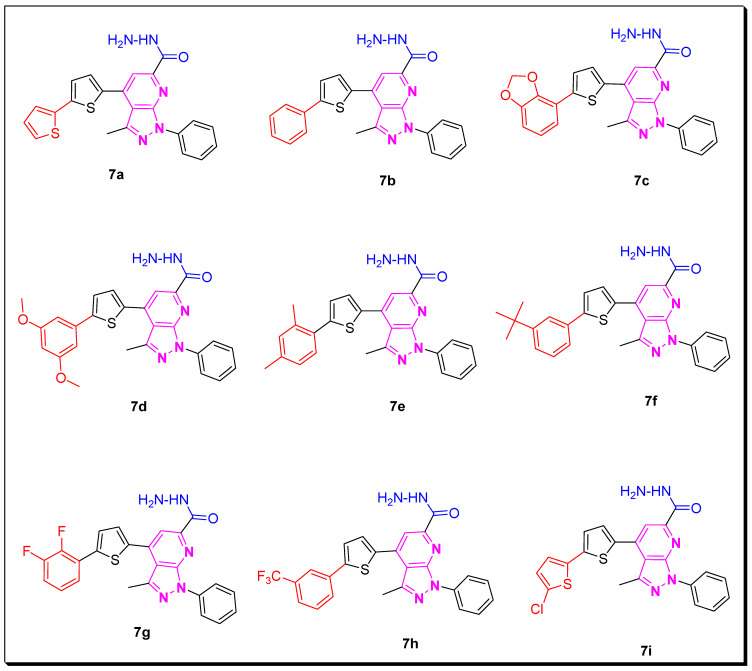
An overview of the aryl-substituted pyrazolo[3,4-*b*]pyridine-6-carbohydrazide derivatives (**7a**–**i**).

The aryl-substituted derivatives (**7a**–**i**) were prepared by refluxing the ethanolic solution of the corresponding substituted biaryl pyrazolo[3,4-*b*]pyridine ester derivatives (**6a**–**i**) (2 mmol) and hydrazine monohydrate (NH_2_NH_2_·H_2_O) (7 mL) for 6–7 h at 79 °C under TLC monitoring. Upon completion of the reaction, the precipitates of (**7a**–**i**) were obtained by cooling the reaction mixture to room temperature, filtered, washed with ethanol (2 × 10 mL) and dried under a vacuum. Further, they were purified by recrystallization using ethanol solvent. The structures of the aryl-substituted pyrazolo[3,4-*b*]pyridine-6-carbohydrazide (**7a**–**i**) derivatives are presented in Figure 8.

### 3.3. Characterization Data

#### 3.3.1. Ethyl 4-(5-Bromothiophen-2-yl)-3-methyl-1-phenyl-1H-pyrazolo[3,4-b]pyridine-6-carboxylate (**4**)

(90%): mp = 203–205 °C: IR (v-cm^−1^); 2353 (C–H), 1705(C=O), 1258 (C–O), 749 (N–H): ^1^H-NMR (400 MHz, DMSO): 7.92 (s, 1H, Py–H), 7.00–8.00 (m, 5H, Ph), 8.30 (d, 2H, CH), 4.54 (q, 2H, OCH_2_), 2.70 (s, 3H, CH_3_), 1.51 (t, 3H, OCH_3_): ^13^C-NMR (126 MHz, DMSO): δ 14.46, 16.08, 62.67, 110.58, 113.85, 112.14, 120.89, 126.43, 128.34, 129.65, 130.38, 135.50, 139.11, 142.87, 144.89, 150.56, 150.99, 165.13: ESI-MS from LC-MS at t = 3.05 min. Calcd. for C_20_H_16_BrN_3_O2S = 441.01, found 442.94.

#### 3.3.2. 4-(5-Bromothiophen-2-yl)-3-methyl-1-phenyl-1H-pyrazolo[3,4-b]pyridine-6-carbohydrazide (**5**)

(86%): mp = 265 °C: IR (v-cm^−1^): 2921 (C–H), 1653 (C=O), 3229 (NH_2_), 666 (NH), 1505 (amide): ^1^H-NMR (400 MHz, DMSO): 10.00 (s, 1H, NH), 7.20–8.00 (m, 5H, Ph), 8.27 (d, 1H, CH), 8.25 (d, 1H, CH) 4.75 (s, 2H, NH_2_), 2.57 (s, 3H, CH_3_):^13^C-NMR (126 MHz, DMSO) δ14.97, 110.47, 112.12, 112.64, 120.62, 126.24, 128.08, 129.69, 130.15, 139.32, 140.09, 143.10, 145.33, 150.48, 150.66, 164.66: ESI-MS from LC-MS at t = 3.36 min. Calcd. for C_18_H_14_BrN_5_OS = 427.01, found 429.9.

#### 3.3.3. Ethyl 4-([2,2′-Bithiophen]-5-yl)-3-methyl-1-phenyl-1H-pyrazolo[3,4-b]pyridine-6-carboxylate (**6a**)

(70%): mp = 154–157 °C: ^1^H-NMR (400 MHz, CDCl_3_): 1.52 (t, 3H, OCH_3_), 2.78 (s, 3H, CH_3_), 4.56 (q, 2H, OCH_2_), 8.01 (s, 1H, Py–H), 7.00–7.80 (m, 5H, Ph), 7.9–8.9 (m, 5H, CH): ^13^C-NMR (101 MHz, CDCl_3_): δ 165.58, 151.64, 151.35, 145.10, 142.89, 139.41, 138.84, 136.87, 134.55, 129.14, 127.95, 125.92, 125.20, 124.37, 123.60, 123.20, 121.20, 113.88, 112.26, 62.40, 16.50, 14.47: ESI-MS from LC-MS at t = 3.79 min. Calcd. for C_24_H_19_N_3_O_2_S_2_ = 445.09, found 446.0.

#### 3.3.4. Ethyl 3-Methyl-1-phenyl-4-(5-phenylthiophen-2-yl)-1H-pyrazolo[3,4-b]pyridine-6-carboxylate (**6b**)

(72%): mp = 171 °C: ^1^H-NMR (400 MHz, CDCl_3_): 1.52 (t, 3H, OCH_3_), 2.78 (s, 3H, CH_3_), 4.56 (q, 2H, OCH_2_), 8.04 (s, 1H, Py–H), 7.00–8.00 (m, 10H, Ph), 8.36 (d, 2H, CH): ^13^C-NMR (101 MHz, CDCl_3_): δ 165.47, 151.53, 144.90, 143.51, 142.76, 139.31, 135.49, 134.37, 129.00, 128.93, 127.53, 126.32, 125.75, 125.44, 123.93, 121.06, 113.78, 112.04, 62.23, 16.38, 14.34: ESI-MS from LC-MS at t = 3.79 min. Calcd. for C_26_H_21_N_3_O_2_S = 439.01, found 440.1.

#### 3.3.5. Ethyl 4-(5-(Benzo[d][1,3]dioxol-4-yl)thiophen-2-yl)-3-methyl-1-phenyl-1H-pyrazolo[3,4-b]pyridine-6-carboxylate (**6c**)

(60%): mp = 127 °C: ^1^H-NMR (400 MHz, CDCl_3_): 1.52 (t, 3H, OCH_3_), 2.78 (s, 3H, CH_3_), 4.55 (q, 2H, OCH_2_), 6.01 (s, 2H, CH_2_), 8.04 (s, 1H, Py–H), 7.00–8.00 (m, 5H, Ph), 8.35 (d, 2H, CH): ^13^C-NMR (101 MHz, CDCl_3_): δ 165.58, 151.62, 148.31, 147.25, 144.89, 143.29, 142.88, 139.44, 134.47, 130.01, 129.12, 125.86, 125.51, 123.13, 121.17, 119.99, 113.88, 112.16, 108.77, 107.04, 101.37, 62.35, 16.50, 14.47: ESI-MS from LC-MS at t = 3.89 min. Calcd. for C_27_H_21_N_3_O_4_S = 483.13, found 484.0.

#### 3.3.6. Ethyl 4-(5-(3,5-Dimethoxyphenyl)thiophene-2-yl)-3-methyl-1-phenyl-1H-pyrazolo[3,4-b]pyridine-6-carboxylate (**6d**)

(68%): mp = 147.5 °C: ^1^H-NMR (400 MHz, CDCl_3_): 1.52 (t, 3H, OCH_3_), 2.78 (s, 3H, CH_3_), 3.88 (s, 6H, CH_6_), 4.55 (q, 2H, OCH_2_), 8.03 (s, 1H, Py–H), 7.00–8.00 (m, 9H, Ph). 8.38 (d, 2H, CH): ^13^C-NMR (101 MHz, CDCl_3_): δ 165.62, 161.34, 151.68, 151.61, 144.95, 143.53, 142.89, 139.43, 137.56, 134.50, 129.13, 125.89, 125.66, 124.44, 121.18, 113.90, 112.17, 104.86, 99.52, 62.39, 55.63, 16.51, 14.47: ESI-MS from LC-MS at t = 3.52 min. Calcd. for C_28_H_25_N_3_O_4_S = 499.58, found 500.0.

#### 3.3.7. Ethyl 4-(5-(3,5-Dimethylphenyl)thiophen-2-yl)-3-methyl-1-phenyl-1H-pyrazolo[3,4-b]pyridine-6-carboxylate (**6e**)

(55%): mp = 137 °C: ^1^H-NMR (400 MHz, CDCl_3_): 1.50 (t, 3H, OCH_3_), 2.38 (s, 3H, CH_3_, CH_3_), 2.78 (s, 3H, CH_3_), 3.88 (s, 6H, CH_6_), 4.55 (q, 2H, OCH_2_), 7.99 (s, 1H, Py–H), 7.00–8.30 (m, 8H, Ph), 8.36 (dd, 2H, CH): ^13^C-NMR (101 MHz, CDCl_3_): δ 165.46, 151.74, 151.62, 143.64, 143.46, 142.75, 139.35, 137.45, 135.49, 134.34, 133.33, 131.35, 129.52, 128.99, 128.40, 126.64, 126.20, 125.71, 121.02, 113.89, 111.95, 62.18, 20.71, 16.36, 14.30. ESI-MS from LC-MS at t = 3.61 min. Calcd. for C_28_H_25_N_3_O_2_S = 467.58, found 468.11.

#### 3.3.8. Ethyl 4-(5-(3-(Tert-butyl)phenyl)thiophen-2-yl)-3-methyl-1-phenyl-1H-pyrazolo[3,4-b]pyridine-6-carboxylate (**6f**)

(69%): mp = 116.3 °C: ^1^H-NMR (400 MHz, CDCl_3_): 1.41 (s, 9H, CH_9_), 1.52 (t, 3H, OCH_3_), 2.79 (s, 3H, CH_3_), 4.56 (q, 2H, OCH_2_), 8.05 (s, 1H, Py–H), 7.00–8.00 (m, 9H, Ph), 8.37 (dd, 2H, CH): ^13^C-NMR (101 MHz, CDCl_3_): δ 165.71, 151.98, 151.75, 151.71, 144.87, 144.29, 142.86, 139.46, 135.41, 134.52, 129.13, 128.78, 125.88, 125.85, 124.80, 124.03, 123.79, 123.52, 121.19, 113.96, 112.11, 62.38, 34.98, 31.56, 16.50, 14.47: ESI-MS from LC-MS at t = 3.70 min. Calcd. for C_30_H_29_N_3_O_2_S = 495.64, found 496.8.

#### 3.3.9. Ethyl 4-(5-(2,3-Difluorophenyl)thiophen-2-yl)-3-methyl-1-phenyl-1H-pyrazolo[3,4-b]pyridine-6-carboxylate (**6g**)

(60%): mp = 170.3 °C: ^1^H-NMR (400 MHz, CDCl_3_): 1.52 (t, 3H, OCH_3_), 2.78 (s, 3H, CH_3_), 4.56 (q, 2H, OCH_2_), 8.02 (s, 1H, Py–H), 7.00–8.00 (m, 8H, Ph), 8.34 (dd, 2H, CH): ^13^C-NMR (101 MHz, CDCl_3_): δ 177.18, 165.54, 151.64, 151.36, 144.78, 142.92, 139.41, 135.93, 134.61, 129.15, 127.81, 127.74, 126.51, 126.48, 125.93, 124.43, 124.02, 121.19, 116.24, 116.07, 113.86, 112.30, 62.40, 16.50, 14.46: ESI-MS from LC-MS at t = 3.70 min. Calcd. for C_30_H_29_N_3_O_2_S = 475.51, found 476.04.

#### 3.3.10. Ethyl 3-Methyl-1-phenyl-4-(5-(3-(trifluoromethyl)phenyl)thiophene-2-yl)-1H-pyrazolo[3,4-b]pyridine-6-carboxylate (**6h**)

(63%): mp = 149.5 °C: ^1^H-NMR (400 MHz, CDCl_3_): 1.53 (t, 3H, OCH_3_), 2.79 (s, 3H, CH_3_), 4.57 (q, 2H, OCH_2_), 8.05 (s, 1H, Py–H), 7.00–8.20 (m, 9H, Ph), 8.35 (dd, 2H, CH): ^13^C-NMR (101 MHz, CDCl_3_): δ 165.57, 151.64, 151.33, 145.71, 142.93, 142.07, 139.39, 136.35, 134.69, 129.57, 129.16, 128.21, 125.97, 125.10, 124.99, 121.23, 113.83, 112.33, 62.44, 16.50, 14.48: ESI-MS from LC-MS at t = 2.85 min. Calcd. for C_27_H_20_F_3_N_3_O_2_S = 507.53, found 508.0.

#### 3.3.11. Ethyl 4-(5′-Chloro-[2,2′-bithiophen]-5-yl)-3-methyl-1-phenyl-1H-pyrazolo[3,4-b]pyridine-6-carboxylate (**6i**)

(70%): mp = 165 °C: ^1^H-NMR (400 MHz, CDCl_3_): 1.52 (t, 3H, OCH_3_), 2.78 (s, 3H, CH_3_), 4.56 (q, 2H, OCH_2_), 7.99 (s, 1H, Py–H), 7.40–7.90 (m, 5H, Ph), 8.34 (dd, 2H, CH), 6.89 (d, 2H, CH), 7.03 (d, 2H, CH): ^13^C-NMR (101 MHz, CDCl_3_): δ 165.53, 151.61, 151.13, 145.44, 142.92, 139.37, 137.46, 136.06, 134.62, 129.15, 128.74, 127.01, 125.97, 124.55, 123.28, 122.74, 121.22, 113.83, 112.35, 62.43, 16.50, 14.47: ESI-MS from LC-MS at t = 9.91 min. Calcd. for C_24_H_18_ClN_3_O_2_S_2_ = 479, found 480.09.

#### 3.3.12. 4-([2,2′-Bithiophen]-5-yl)-3-methyl-1-phenyl-1H-pyrazolo[3,4-b]pyridine-6-carbohydrazide (**7a**)

(80%): mp = 255 °C: ^1^H-NMR (400 MHz, DMSO): 2.58 (s, 3H, CH_3_), 4.75 (s, 2H, NH_2_), 8.04 (s, 1H, Py–H), 7.00–7.80 (m, 5H, Ph), 7.9–8.9 (m, 5H, CH), 10.04 (s, 1H, NH): ^13^C-NMR (101 MHz, DMSO): δ 164.42, 151.45, 150.37, 144.40, 142.63, 142.55, 139.45, 139.00, 134.69, 129.23, 128.95, 127.48, 126.55, 125.93, 125.67, 124.84, 120.09, 111.77, 111.71, 14.27: ESI-MS from LC-MS at t = 3.79 min. Calcd. for C_22_H_17_N_5_OS_2_ = 471.53, found 472.0.

#### 3.3.13. 3-Methyl-1-phenyl-4-(5-phenylthiophen-2-yl)-1H-pyrazolo[3,4-b]pyridine-6-carbohydrazide (**7b**)

(81%): mp = 265 °C: ^1^H-NMR (400 MHz, DMSO): 2.58 (s, 3H, CH_3_), 4.76 (s, 2H, NH_2_), 8.07 (s, 1H, Py–H), 7.00–8.50 (m, 10H, Ph), 8.23 (dd, 2H, CH) 10.03 (s, 1H, NH): ^13^C-NMR (101 MHz, DMSO): δ 164.89, 151.92, 150.84, 144.87, 143.10, 143.02, 139.92, 139.47, 135.16, 129.70, 129.42, 127.95, 127.02, 126.40, 126.14, 125.31, 120.56, 112.24, 112.18, 14.74: ESI-MS from LC-MS at t = 2.85 min. Calcd. for C_24_H_19_N_5_OS = 425.51, found 425 + 41 = 466. M.W of ACN = 41.

#### 3.3.14. 4-(5-(Benzo[d][1,3]dioxol-4-yl)thiophen-2-yl)-3-methyl-1-phenyl-1H-pyrazolo[3,4-b]pyridine-6-carbohydrazide (**7c**)

(70%): mp = 276 °C: ^1^H-NMR (400 MHz, DMSO): 2.57 (s, 3H, CH_3_), 4.75 (s, 2H, NH_2_), 6.07 (s, 2H, CH_2_), 8.05 (s, 1H, Py–H), 7.00–8.20 (m, 10H, Ph), 8.31 (dd, 2H, CH), 10.02 (s, 1H, NH): ^13^C-NMR (101 MHz, DMSO): δ 164.91, 151.95, 150.83, 148.42, 147.14, 144.61, 143.00, 142.82, 139.95, 139.47, 129.69, 129.53, 126.12, 120.53, 120.00, 112.20, 109.09, 106.96, 101.62, 79.65, 14.70: ESI-MS from LC-MS at t = 3.70 min. Calcd. for C_25_H_19_N_5_O_3_S = 469.52, found 469 + 41 = 510. M.W of acetonitrile = 41.

#### 3.3.15. 4-(5-(3,5-Dimethoxyphenyl)thiophen-2-yl)-3-methyl-1-phenyl-1H-pyrazolo[3,4-b]pyridine-6-carbohydrazide (**7d**)

(83%): mp = 249 °C: ^1^H-NMR (400 MHz, DMSO): 2.32 (s, 3H, CH_3_), 2.37 (s, 3H, CH_3_), 2.58 (s, 3H, CH_3_), 4.73 (s, 2H, NH_2_), 7.98 (s, 1H, Py–H), 7.00–8.50 (m, 10H, Ph), 8.32 (m, 2H, CH), 10.01 (s, 1H, NH): ^13^C-NMR (101 MHz, DMSO): δ 164.84, 151.99, 150.90, 143.69, 143.36, 143.04, 139.85, 139.48, 137.19, 135.27, 133.21, 131.72, 129.77, 129.68, 127.51, 127.09, 126.12, 120.54, 112.16, 21.04, 14.80: ESI-MS from LC-MS at t = 3.92 min. Calcd. for C_26_H_23_N_5_O_3_S = 485.56, found 486.0 + 41 = 510. M.W of acetonitrile = 41.

#### 3.3.16. 4-(5-(2,4-Dimethylphenyl)thiophen-2-yl)-3-methyl-1-phenyl-1H-pyrazolo[3,4-b]pyridine-6-carbohydrazide (**7e**)

(85%): mp = 290 °C: ^1^H-NMR (400 MHz, DMSO): 2.32 (s, 3H, CH_3_), 2.37 (s, 3H, CH_3_), 2.57 (s, 3H, CH_3_), 4.73 (s, 2H, NH_2_), 7.98 (s, 1H, Py–H), 7.00–8.20 (m, 8H, Ph), 8.32 (m, 2H, CH), 10.01 (s, 1H, NH): ^13^C-NMR (101 MHz, DMSO): δ 164.42, 151.57, 150.48, 143.27, 142.94, 142.62, 139.42, 139.06, 136.77, 134.85, 132.78, 131.30, 129.35, 129.26, 127.09, 126.67, 125.70, 120.12, 111.77, 20.72, 20.62, 14.38: ESI-MS from LC-MS at t = 3.93 min. Calcd. for C_26_H_23_N_5_OS = 453.56, found 454.09.

#### 3.3.17. 4-(5-(3-(Tert-butyl)phenyl)thiophen-2-yl)-3-methyl-1-phenyl-1H-pyrazolo[3,4-b]pyridine-6-carbohydrazide (**7f**)

(76%): ^1^H-NMR (400 MHz, CDCl_3_): 1.41 (s, 9H, CH_9_), 2.79 (s, 3H, CH_3_), 4.56 (s, 2H, NH_2_), 8.05 (s, 1H, Py–H), 7.00–8.00 (m, 11H, Ph), 8.30–8.90 (m, 2H, CH), 10.01 (s, 1H, NH). ^13^C-NMR (101 MHz, DMSO): δ 164.42, 151.57, 150.48, 143.27, 142.94, 142.62, 139.42, 139.06, 136.77, 134.85, 132.78, 131.30, 129.35, 129.26, 127.09, 126.67, 125.70, 120.12, 111.77, 20.72, 20.62, 14.38: ESI-MS from LC-MS at t = 3.70 min. Calcd. for C_28_H_27_N_5_OS = 467.58, found 510.0.

#### 3.3.18. 4-(5-(2,3-Fluorophenyl)thiophen-2-yl)-3-methyl-1-phenyl-1H-pyrazolo[3,4-b]pyridine-6-carbohydrazide (**7g**)

(71%): mp = 275 °C: ^1^H-NMR (400 MHz, DMSO): 2.57 (s, 3H, CH_3_), 4.74 (s, 2H, NH_2_), 8.01 (s, 1H, Py–H), 7.00–8.50 (m, 5H, Ph), 8.20–8.90 (m, 2H, CH), 10.01 (s, 1H, NH): ^13^C-NMR (101 MHz, DMSO): δ 164.36, 150.89, 150.40, 150.31, 147.21, 144.60, 143.41, 142.51, 139.42, 138.96, 138.20, 136.62, 135.84, 129.24, 126.49, 125.75, 123.91, 123.04, 120.12, 14.28: ESI-MS from LC-MS at t = 4.28 min. Calcd. for C_24_H_17_F_2_N_5_OS = 461.49, found 462.05.

#### 3.3.19. 3-Methyl-1-phenyl-4-(5-(3-(trifluoromethyl)phenyl)thiophen-2-yl)-1H-pyrazolo[3,4-b]pyridine-6-carbohydrazide (**7h**)

(65%): mp = 278 °C: ^1^H-NMR (400 MHz, DMSO): 2.58 (s, 3H, CH_3_), 4.77 (s, 2H, NH_2_), 8.11 (s, 1H, Py–H), 7.00–8.50 (m, 9H, Ph), 8.29 (m, 2H, CH), 10.06 (s, 1H, NH): ^13^C-NMR (101 MHz, DMSO): δ 164.89, 151.76, 150.77, 145.28, 143.01, 141.35, 140.03, 139.43, 136.09, 130.50, 130.28, 130.20, 129.71, 126.98, 126.79, 126.18, 123.38, 120.57, 112.31, 112.19, 14.64: ESI-MS from LC-MS at t = 3.74 min. Calcd. for C_25_H_18_F_3_N_5_OS = 493.50, found 494.03.

#### 3.3.20. 4-(5′-Chloro-[2,2′-bithiophen]-5-yl)-3-methyl-1-phenyl-1H-pyrazolo[3,4-b]pyridine-6-carbohydrazide (**7i**)

(79%): mp = 270 °C: ^1^H-NMR (400 MHz, DMSO): 2.57 (s, 3H, CH_3_), 4.76 (s, 2H, NH_2_), 8.01 (s, 1H, H), 7.00–8.50 (m, 5H, Ph), 7.95–8.32 (m, 4H, CH), Py- 10.00 (s, 1H, NH): ^13^C-NMR (101 MHz, DMSO): δ 164.80, 151.40, 150.75, 145.32, 143.04, 139.40, 137.68, 135.77, 129.70, 128.40, 127.09, 126.17, 124.25, 120.59, 112.41, 112.15, 14.71: ESI-MS from LC-MS at t = 3.63 min. Calcd. for C_22_H_16_ClN_5_OS_2_ = 465.98, found 466.1 + 41 = 510. M.W of acetonitrile = 41.

### 3.4. Procedure for Anti-Diabetic Activity Analysis

The anti-diabetic activity of all the prepared derivatives (**6a**–**i**) and (**7a**–**i**) was evaluated following the method reported in refs. [31,32]. In brief, to check the anti-diabetic activity of each compound, six different concentrations, ranging from 250 to 50 μg, with a difference of 50 μg, were used and each concentration was treated with 200 μL of α-amylase (Hi media RM 638) enzyme to determine the anti-diabetic activity in triplicate at each concentration. Here, 0.6 mL of each sample at each concentration was mixed with 0.6 mL of buffered solution (0.03 M sodium phosphate buffer), which was subsequently incubated for 16 min at 37 °C. Afterwards, 1 mL of starch solution was added to the above solutions. In the next step, 1 mL of color-imparting reagent, 3,6-dinitrosalicylic acid (DNSA), was poured into each solution and the solution was left for 6 min in boiling water. Finally, the solutions were made up to 20 mL with distilled water. Acarbose and control (i.e., without any sample) were used as a standard and blank, respectively. The absorbance value taken at 670 nm was used to check the performance of the newly obtained derivatives. The following Equation (1) was used to determine the percentage inhibition of each drug at the different concentrations.

(1) $$\%A=\frac{A_{control}-A_{sample}}{A_{control}}\times 100$$

where *%A*, *A_control_* and *A_sample_* are the percentage inhibition, absorbance blank and absorbance of sample, respectively.

The concentration of pyrazolo[3,4-*b*]pyridine derivative needed to inhibit the α-amylase activity by 50% is known as the IC_50_ value and is calculated from the plot of the percent inhibition against the log inhibitor concentration (i.e., synthesized drugs). A non-linear regression analysis on Graph Pad Prism 8.0.2. was performed on the plots to extract the IC_50_ value for each compound.

### 3.5. Molecular Docking Studies

The newly prepared ester (**6a**–**i**) and hydrazide (**7a**–**i**) derivatives were further modelled for in silico molecular docking of the diabetic enzyme α-amylase. The chemical structures of all the synthesized compounds were drawn using ChemDraw software (12.0.2.1076), and the conformation of the biaryl derivatives (**6a**–**i**) and (**7a**–**i**) was optimized by minimizing its energy using the Avogadro software (1.2.0). The default force field applied was the universal force field (UFF). The chemical structures with stable conformation were saved in the PDB format. Later, these optimized PDB files were employed to perform the docking studies. The PubChem database was used to fetch the 3D structure of acarbose in PDB format and used as the reference drug for the α-amylase inhibitor. The three-dimensional (3D) structure of the receptor, i.e., alpha-amylase (PDB ID: 3bc9; 1.36 Å), was retrieved from the Protein Data Bank [33]. The preparation of the receptor was performed using Autodock 1.6.6. tool, which includes various steps, such as the removal of water molecules, the addition of polar hydrogen atoms and Kollman charges and the selection of the AD_7_ type of atoms. The grid box with XYZ dimensions of (126 × 126 × 126) and spacing of 0.66 Å was used to identify the active sites of the α-amylase enzyme [34]. Afterward, the tested derivatives were docked within the active sites of the α-amylase enzyme to evaluate their interactions and biding affinity with the active site in order to establish the relationship between different amino acids, the binding affinities with ligands and their role in the corresponding anti-diabetic activity. The two-dimensional (2D) and three-dimensional (3D) structures were acquired via Discovery Studio 2020 Client.

## 4. Conclusions

In this study, a series of eighteen different biaryl pyrazolo[3,4-*b*]pyridine ester (**6a**–**i**) and hydrazide derivatives (**7a**–**i**) were synthesized via the Suzuki cross-coupling reaction. The synthesis involved initially creating Suzuki ester derivatives, which were then transformed into the corresponding hydrazide derivatives. The reverse synthesis route proved unsuccessful. The synthesized esters (**6a**–**i**) and hydrazides (**7a**–**i**) were screened for anti-diabetic activities against the α-amylase enzyme. Among the carboxylate derivatives (**6a**–**i**), the compounds **6b**, **6c**, **6h**, and **6g** exhibited excellent inhibition, with IC_50_ values of 5.14, 5.15, 5.56, and 5.20 μM, respectively, displaying higher anti-diabetic inhibitory activity compared to the reference drug acarbose (IC_50_ = 200.1 ± 1.00 μM). These compounds demonstrated better binding scores: **6b** (−7.33 kcal mol^−1^), **6c** (−6.97 kcal mol^−1^), **6h** (−6.67 kcal mol^−1^), and **6g** (−6.92 kcal mol^−1^) than acarbose (−5.57 kcal mol^−1^). Similarly, in the hydrazide series (**7a**–**i**), the newly synthesized derivatives, such as **7a**, **7b**, **7c**, **7d**, **7f**, **7g**, and **7h**, exhibited excellent in vitro anti-diabetic activities of 5.21, 5.18, 5.17, 5.12, 5.10, 5.16, and 6.19 μM, respectively. These compounds also displayed better binding scores: **7a** (−8.13 kcal mol^−1^), **7b** (−8.68 kcal mol^−1^), **7c** (−8.71 kcal mol^−1^), **7d** (−8.61 kcal mol^−1^), **7f** (−8.73 kcal mol^−1^), **7g** (−8.77 kcal mol^−1^), and **7h** (−8.18 kcal mol^−1^) in comparison to the reference standard drug acarbose (−5.57 kcal mol^−1^). The derivative **6b** with a phenyl ring attached to the fourth position of the thiophene ring, and compound **6c**, featuring a benzo-1,3-dioxolane at the same position, displayed excellent alpha-amylase inhibitory efficacy, with IC_50_ values of 5.14 and 5.15 µM, respectively. The hydrazide compound **7f** had the highest chemotherapeutic potential among both ester and hydrazide series, which is attributed to the tertiary butyl functional on the phenyl ring attached to the thiophene and the hydrazide moieties. The molecular docking studies were in agreement with and supported the in vitro anti-diabetic assay results, suggesting that these pyrazolo[3,4-*b*]pyridine compounds could serve as α-amylase enzyme inhibitors, and further, that these compounds should be tested for in vivo models and ADMET studies.

## Figures and Tables

**Table 1:** The detailed study of the effect of different reaction parameters, such as the palladium catalyst, and solvents and base, on the percentage yield of the Suzuki cross-coupling reaction.

Entry	Catalyst	Base	Solvent	% Yield
**1**	Pd(PPh_3_)_2_	K_2_CO_3_	Dioxane/water	70
			Toluene/water	16
			Dioxane	Unsuccessful
**2**	Pd(PPh_3_)_2_	K_2_CO_3_	Dioxane/water	50
	Pd(dppf_3_)_2_			Traces
	Cu(OAC)_2_			Unsuccessful
**3**	Pd(PPh_3_)_2_	CH_3_CO_2_K	Dioxane/water	10
		K_2_CO_3_		66

**Table 2:** In vitro anti-diabetic IC_50_ values of the synthesized heteroaryl ester derivatives (**6a**–**i**). In table, A represents the acarbose standard.

Compound	IC_50_ Value (µm)
**4**	7.26
**6a**	8.38
**6b**	5.14
**6c**	5.15
**6d**	7.06
**6e**	9.01
**6f**	58.56
**6g**	5.56
**6h**	5.20
**6i**	7.40
**Acarbose**	200.1

**Table 3:** Anti-diabetic IC_50_ values of synthesized heteroaryl hydrazide derivatives (**7a–i**). In Table 3 is the hydrazide and A is the acarbose standard drug.

Compound	IC_50_ Value (µM)
**5**	7.91
**7a**	5.21
**7b**	5.18
**7c**	5.17
**7d**	5.12
**7e**	8.94
**7f**	5.10
**7g**	5.15
**7h**	5.19
**7i**	8.45
**Acarbose**	200.1

**Table 4:** Binding energy (∆G) values of the hetero aryl carboxylate (**6a**–**i**) and hydrazide (**7a**–**i**) derivatives.

Ester Derivatives	∆G (kcal/mol)	Hydrazide Derivatives	∆G (kcal/mol)
		**5**	−6.70
**6a**	−6.87	**7a**	−8.13
**6b**	−7.33	**7b**	−8.68
**6c**	−6.97	**7c**	−8.71
**6d**	−6.87	**7d**	−8.61
**6e**	−6.07	**7e**	−7.31
**6f**	−6.8	**7f**	−8.73
**6g**	−6.67	**7g**	−8.77
**6h**	−6.92	**7h**	−8.18
**6i**	−6.06	**7i**	−7.92
**Acarbose**	−5.57		

**Table 5:** The ∆G (kcal/mol) values and type of interaction of a potent pyrazolo[3,4-*b*]pyridine-6-carboxylates derivative with specific residues of the α-amylase enzyme.

Ligand (∆G)	Type of Bonding	Interacting Amino Acids
**6b** (−7.33)	H-bonding, π–donor H-bonding *, π alkyl	LYS A:227, ARG A:373, ARG A:373, ASP A:373
**6c** (−6.97)	H-bonding, π–alkyl, alkyl	LYS A:227, ARG A:373, GLN A:119, ARG A:373, ASN A:372
**6g** (−6.67)	H-bonding, π-sigma,	HIS A:123, LYS A:227, ARG A:373, HIS A:219
**6h** (−6.92)	Fluorine, π–sigma, amide–π–π stacked, π–alkyl	GLU A:168, GLU A:167, PRO A:167, ALA A:163,

* Hydrogen bonding.

**Table 6:** The ∆G values along with the type of bonding and interacting residues of the pyrazolo[3,4-*b*]pyridine-6-carbohydrazide derivatives (**7a**–**i**).

Ligand (∆G)	Type of Bonding	Interacting Amino Acids
**7a** (−8.13)	H-bonding, π–alkyl	ASN A:112, TYR A:507, GLN A:110, PRO A:82, ALA A:631
**7b** (−8.68)	H-bonding, π–alkyl, π–π stacked	ACN A:302, THR A:307, TPR A:310, TPR A:306
**7c** (−8.71)	π–alkyl, π–π stacked, attractive charge	TPR A:306, TPR A:310, TPR A:307, GLU A:323
**7d** (−8.61)	H-bonding, attractive charge, carbon–hydrogen bond	ACN A:309, THR A:307, ASP A:311, ASP A:303, TRP A:307
**7f** (−8.73)	H-bonding, attractive charge, π–π stacked	THR A:307, TYR A:367, TRP A:310, TRP A:306, TRP A:307, LEU A:298
**7g** (−8.77)	H-bonding, π–π stacked	THR A:307,ASN A:309, TPR A:306, TPR A:310
**7h** (−8.18)	H-bonding, π–sulfur, halogen bonding, π–π T-shaped	THR A:307, TYR A:308, TPR A:310, ASN A:278, ASP A:311, TPR A:306

## Data Availability

Data is contained within the article and Supplementary Material. Further data related to this study can be obtained on reasonable request from corresponding author Tahir Maqbool.

## Supplementary Materials

The following supporting information can be downloaded at: https://www.mdpi.com/article/10.3390/ph17101326/s1, Figure S1. Mass spectrum of 4, Figure S2. 1H-NMR spectrum of 4, Figure S3. 13C-NMR spectrum of 4, Figure S4. Mass spectrum of 5, Figure S5. 1H-NMR spectrum of 3, Figure S6. 13C-NMR spectrum of 5, Figure S7. Mass spectrum of 6a, Figure S8. 1H-NMR spectrum of 6a, Figure S9. 13C-NMR spectrum of 6a, Figure S10. Mass spectrum of 6b, Figure S11. 1H-NMR spectrum of 6b, Figure S12. 13C-NMR spectrum of 6b, Figure S13. Mass spectrum of 6c, Figure S14. 1H-NMR spectrum of 6c, Figure S15. 13C-NMR spectrum of 6c, Figure S16. Mass spectrum of 6d, Figure S17. 1H-NMR spectrum of 6d, Figure S18. 1H-NMR spectrum of 6d, Figure S19. Mass spectrum of 6e, Figure S20. 1H-NMR spectrum of 6e, Figure S21. 13C-NMR spectrum of 6e, Figure S22. Mass spectrum of 6f, Figure S23. 1H-NMR spectrum of 6f, Figure S24. 13C-NMR spectrum of 6f, Figure S25. Mass spectrum of 6g, Figure S26. 1H-NMR spectrum of 6g, Figure S27. 13C-NMR spectrum of 6g, Figure S28. Mass spectrum of 6h, Figure S29. 1H-NMR spectrum of 6h, Figure S30. 13C-NMR spectrum of 6h, Figure S31. Mass spectrum of 6i, Figure S32. 1H-NMR spectrum of 6i, Figure S33. 13C-NMR spectrum of 6i, Figure S34. Mass spectrum of 7a, Figure S35. 1H-NMR spectrum of 7a, Figure S36. 13C-NMR spectrum of 7a, Figure S37. Mass spectrum of 7b, Figure S38. 1H-NMR spectrum of 7b, Figure S39. 13C-NMR spectrum of 7b, Figure S40. Mass spectrum of 7c, Figure S41. 1H-NMR spectrum of 7c, Figure S42. 13C-NMR spectrum of 7c, Figure S43. Mass spectrum of 7d, Figure S44. 1H-NMR spectrum of 7d, Figure S45. 13C-NMR spectrum of 7d, Figure S46. Mass spectrum of 7e, Figure S47. 1H-NMR spectrum of 7e, Figure S48. 13C-NMR spectrum of 7e, Figure S49. Mass spectrum of 7f, Figure S50. 1H-NMR spectrum of 7f, Figure S51. 13C-NMR spectrum of 7f, Figure S52. Mass spectrum of 7g, Figure S53. 1H-NMR spectrum of 7g, Figure S54. 13C-NMR spectrum of 7g, Figure S55. Mass spectrum of 7h, Figure S56. 1H-NMR spectrum of 7h, Figure S57. 13C-NMR spectrum of 7h, Figure S58. Mass spectrum of 7i, Figure S59. 1H-NMR spectrum of 7i. Figure S60. 13C-NMR spectrum of 7i.
