# Supplementary material for: Anti-Diabetic Activities and Molecular Docking Studies of Aryl-Substituted Pyrazolo[3,4-b]pyridine Derivatives Synthesized via Suzuki Cross-Coupling Reaction
Source: Pharmaceuticals (Basel). 2024 Oct 4;17(10):1326. doi: 10.3390/ph17101326 (PMC11510069; doi:10.3390/ph17101326)
Supplement: Supplementary file 1 [file pharmaceuticals-17-01326-s001.zip › pharmaceuticals-3176315-supplementary.pdf]

**Electronic Supplementary Information**

# Anti-Diabetic Activities and Molecular Docking Studies of Aryl-Substituted Pyrazolo[3,4-b]pyridine Derivatives Synthesized via Suzuki Cross-Coupling Reaction

Iqra Rafique[1,2], Tahir Maqbool*[1], Floris P.J.T. Rutjes[2], Ali Irfan[1], Yousef A. Bin Jardan[3]**

[1]Department of chemistry, Government college University Faisalabad, Faisalabad 38000, Pakistan. iqrarafique298@gmail.com (I.R.); drtahirmaqbool@gcuf.edu.pk (T.M.); raialiirfan@gmail.com (A.I)

[2]Synthetic Organic Chemistry (SOC) Group, Radboud University, 6525 AJ Nijmegen, Netherland. floris.rutjes@ru.nl (F.P.J.T)

[3] Department of Pharmaceutics, College of Pharmacy, King Saud University, Riyadh 11451, Saudi Arabia. ybinjardan@ksu.edu.sa (Y.B.J)

**Corresponding authors**: drtahirmaqbool@gcuf.edu.pk (T.M.); ybinjardan@ksu.edu.sa (Y.B.J)

**Ethyl 4-(5-bromothiophen-2-yl)-3-methyl-1-phenyl-1*H*-pyrazolo[3,4-*b*]pyridine-6-carboxylate (4)**

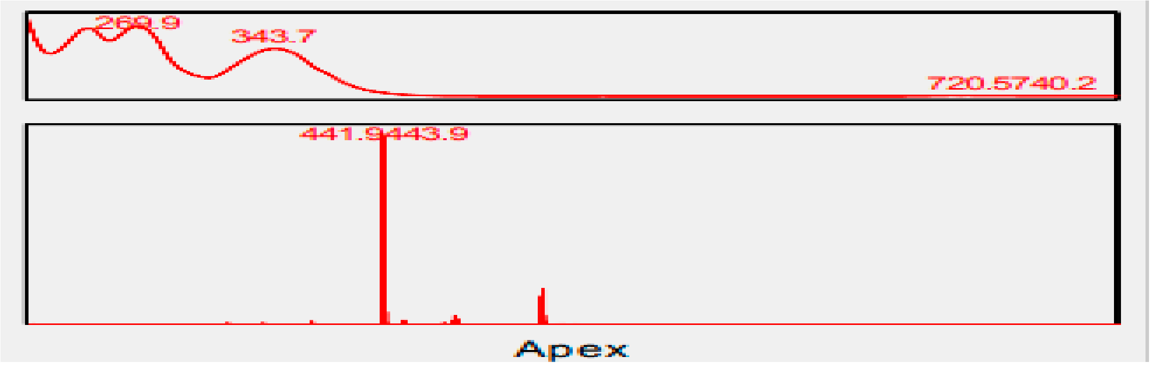


**Figure S1.** Mass spectrum of 4.

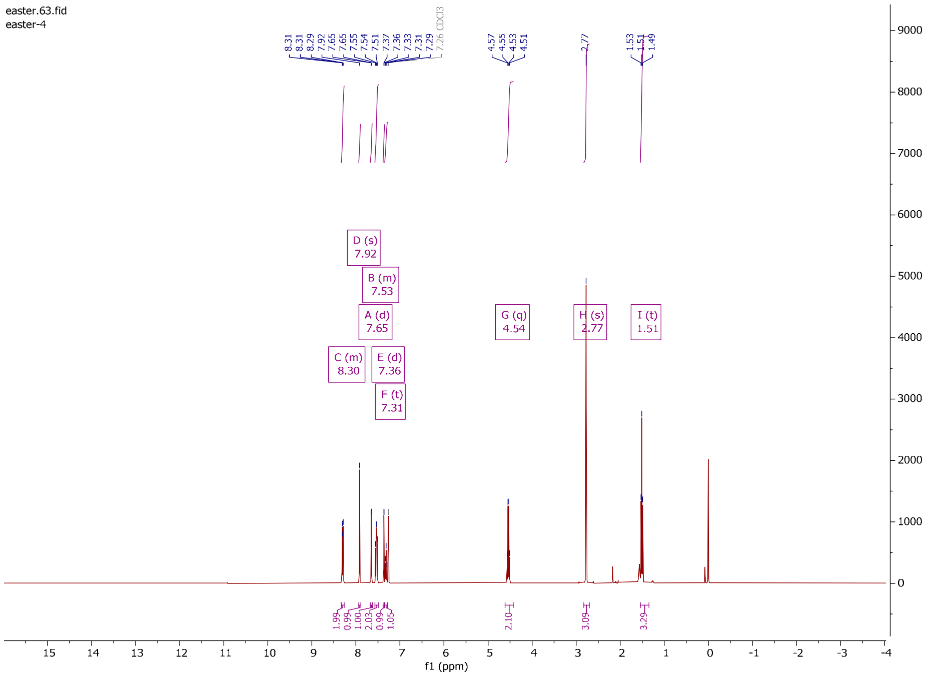


**Figure S2.** $^{1}$H-NMR spectrum of 4.

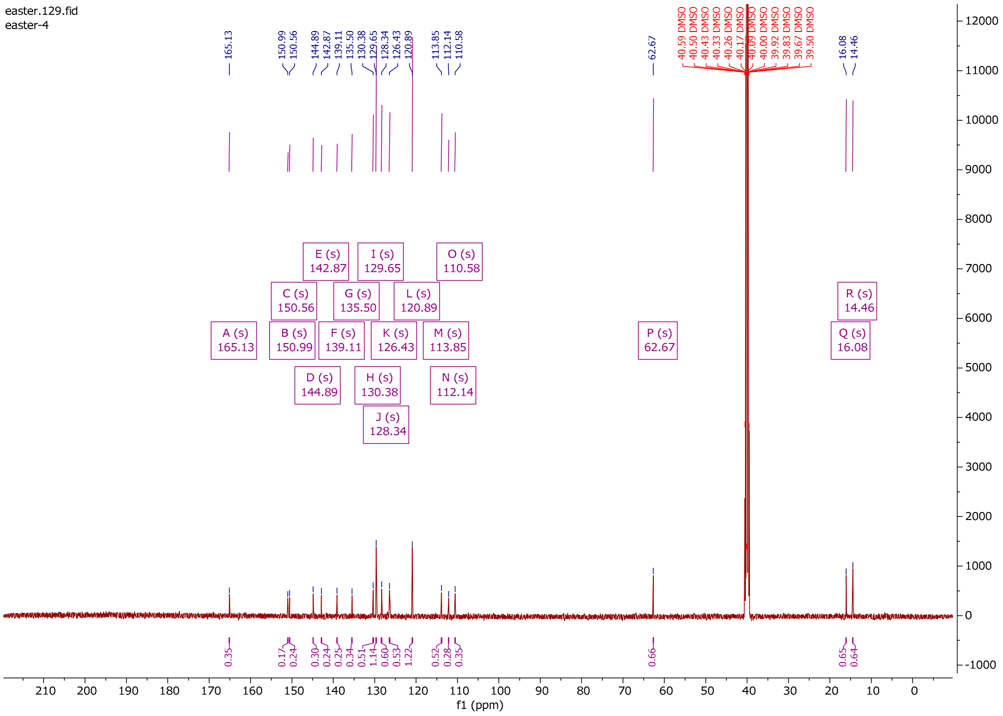


**Figure S3.** $^{13}$C-NMR spectrum of 4.

**4-(5-bromothiophen-2-yl)-3-methyl-1-phenyl-1*H*-pyrazolo[3,4-*b*]pyridine-6-carbohydrazide (5)**

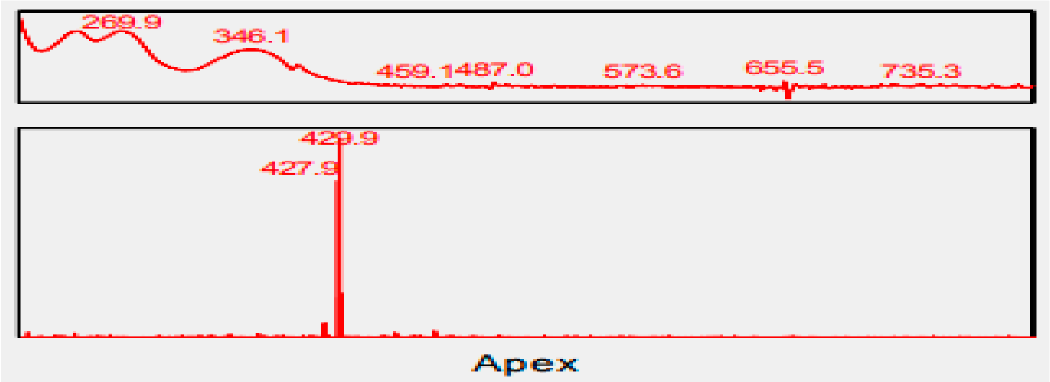


**Figure S4.** Mass spectrum of 5.

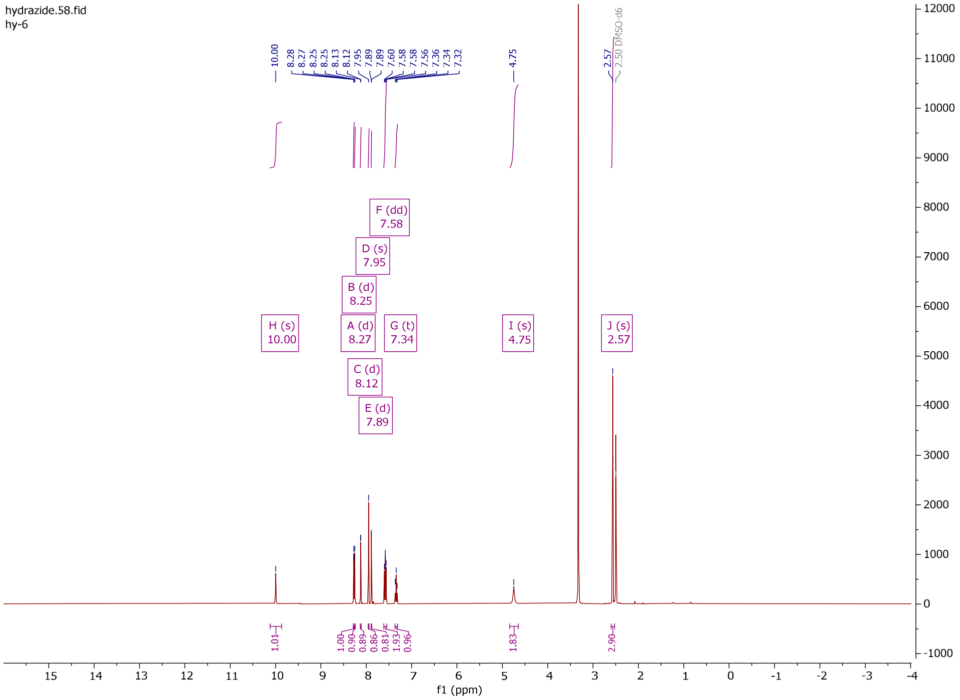


**Figure S5.** $^{1}$H-NMR spectrum of 3.

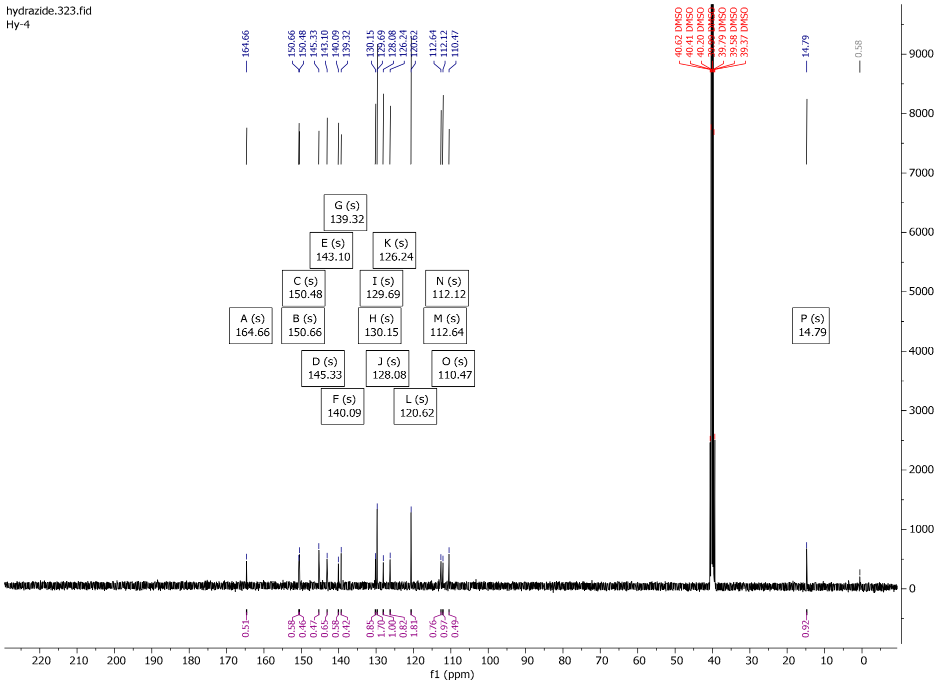


**Figure S6.** $^{13}$C-NMR spectrum of 5.

**Ethyl 4-([2,2'-bithiophen]-5-yl)-3-methyl-1-phenyl-1*H*-pyrazolo[3,4-*b*]pyridine-6-carboxylate (6a)**

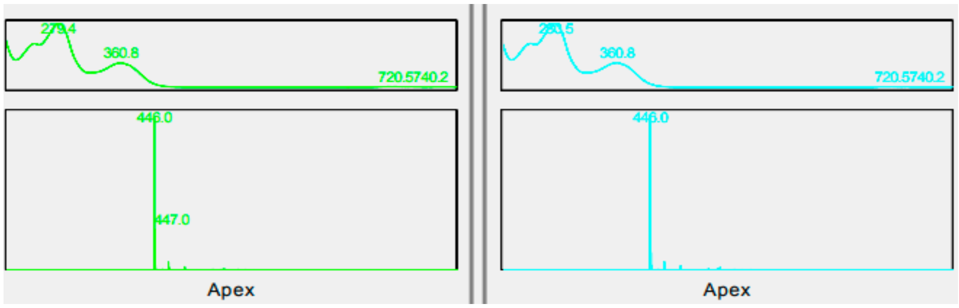


**Figure S7.** Mass spectrum of 6a.

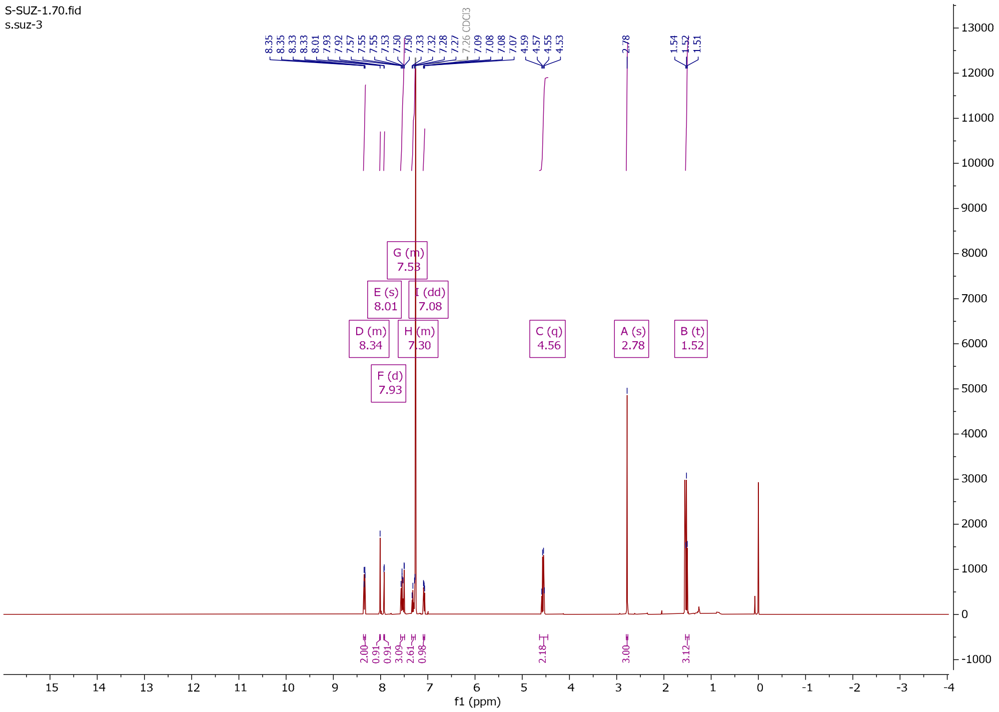


**Figure S8.** $^{1}$H-NMR spectrum of 6a.

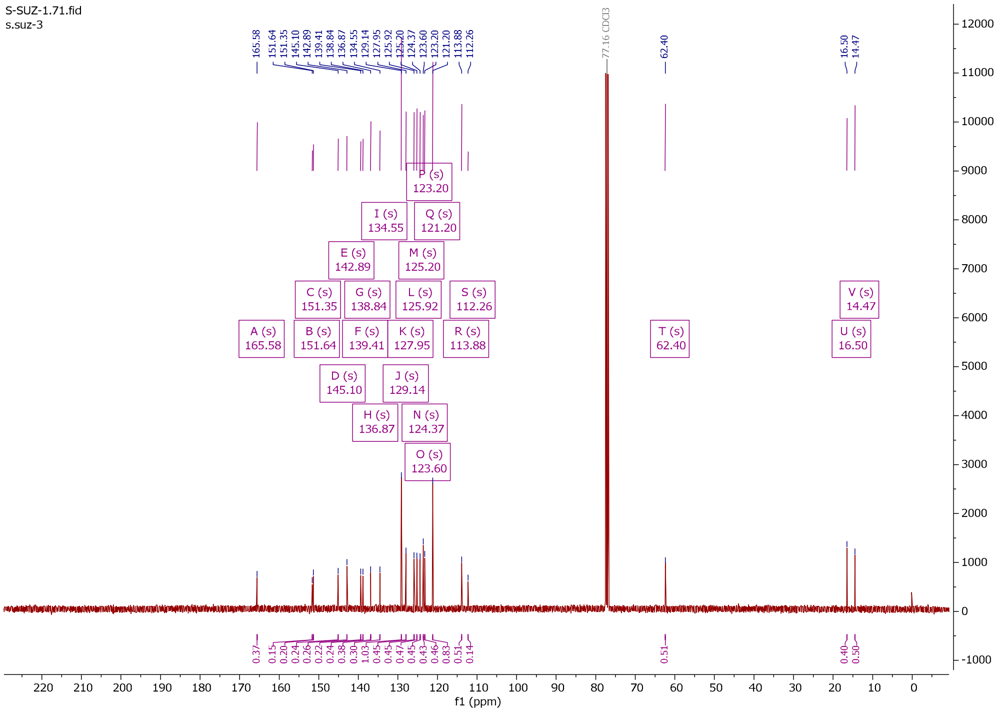


**Figure S9.** $^{13}$C-NMR spectrum of 6a.

**Ethyl 3-methyl-1-phenyl-4-(5-phenylthiophen-2-yl)-1*H*-pyrazolo[3,4-*b*]pyridine-6-carboxylate (6b)**

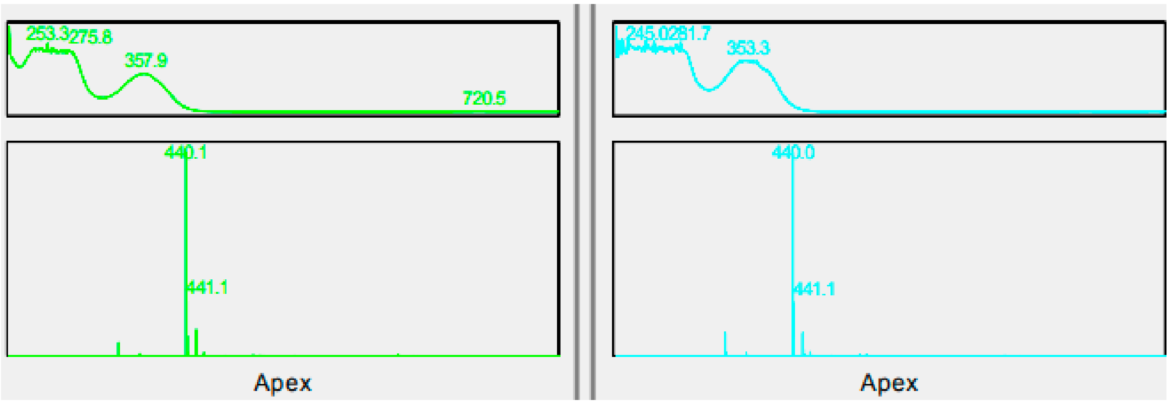


**Figure S10.** Mass spectrum of 6b.

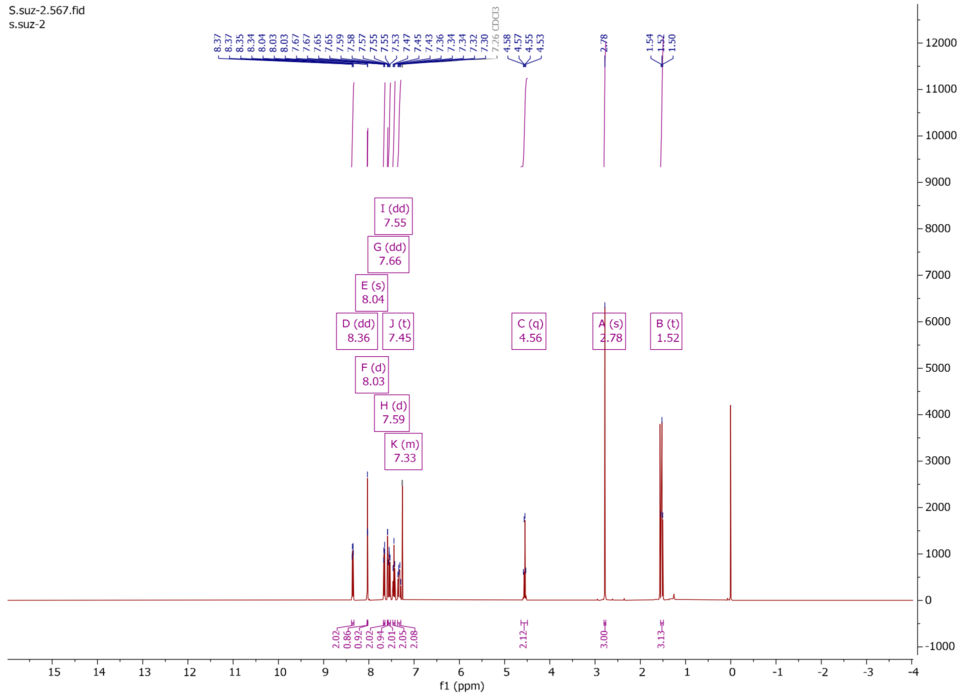

**Figure S11.** $^{1}$H-NMR spectrum of 6b.

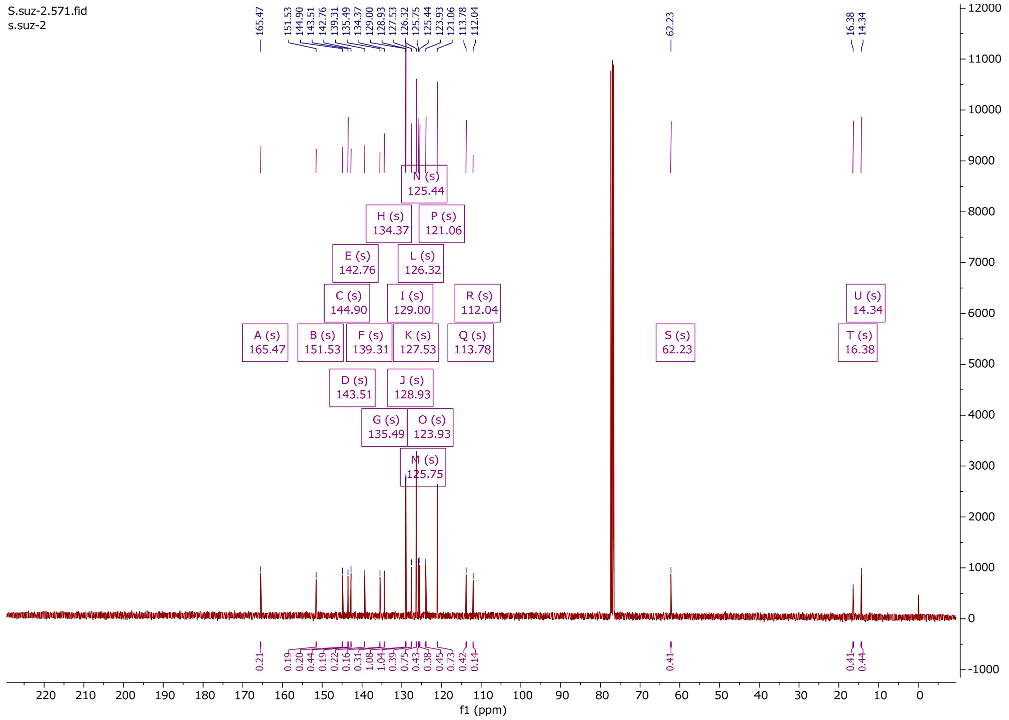

**Figure S12.** $^{13}$C-NMR spectrum of 6b.

**Ethyl 4-(5-(benzo[d][1,3]dioxol-4-yl)thiophen-2-yl)-3-methyl-1-phenyl-1*H*-pyrazolo[3,4-*b*]pyridine-6-carboxylate (6c)**

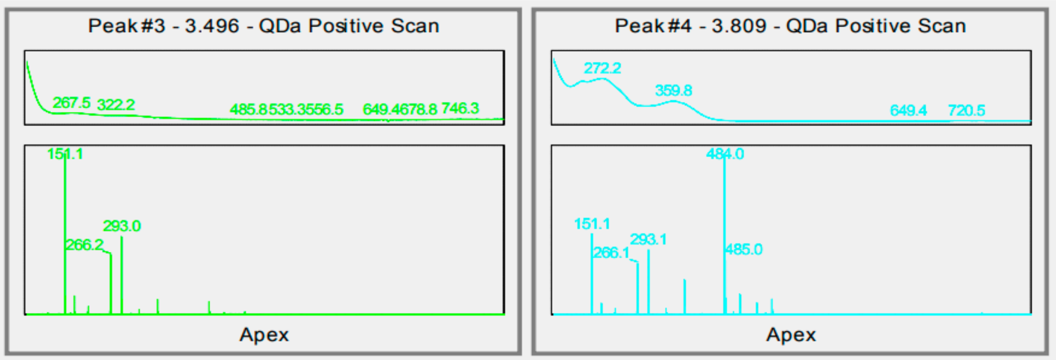


**Figure S13.** Mass spectrum of 6c.

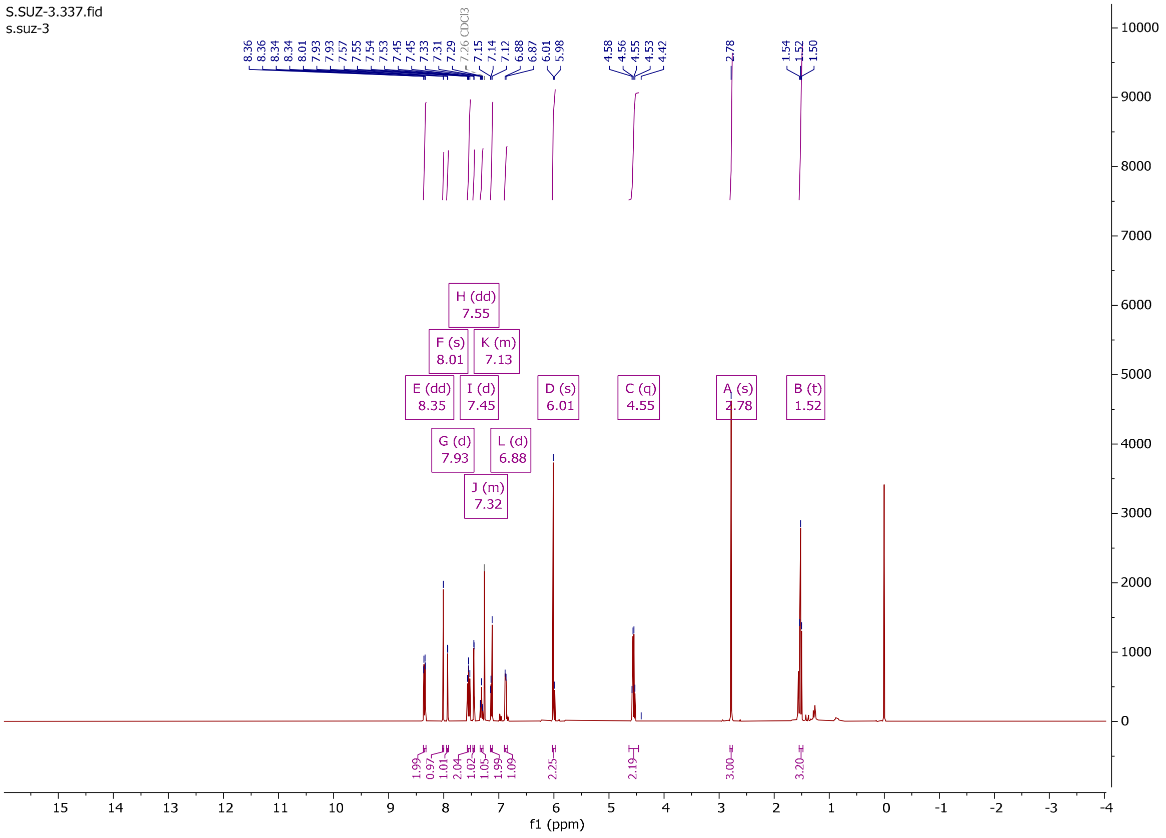


**Figure S14.** $^{1}$H-NMR spectrum of 6c.

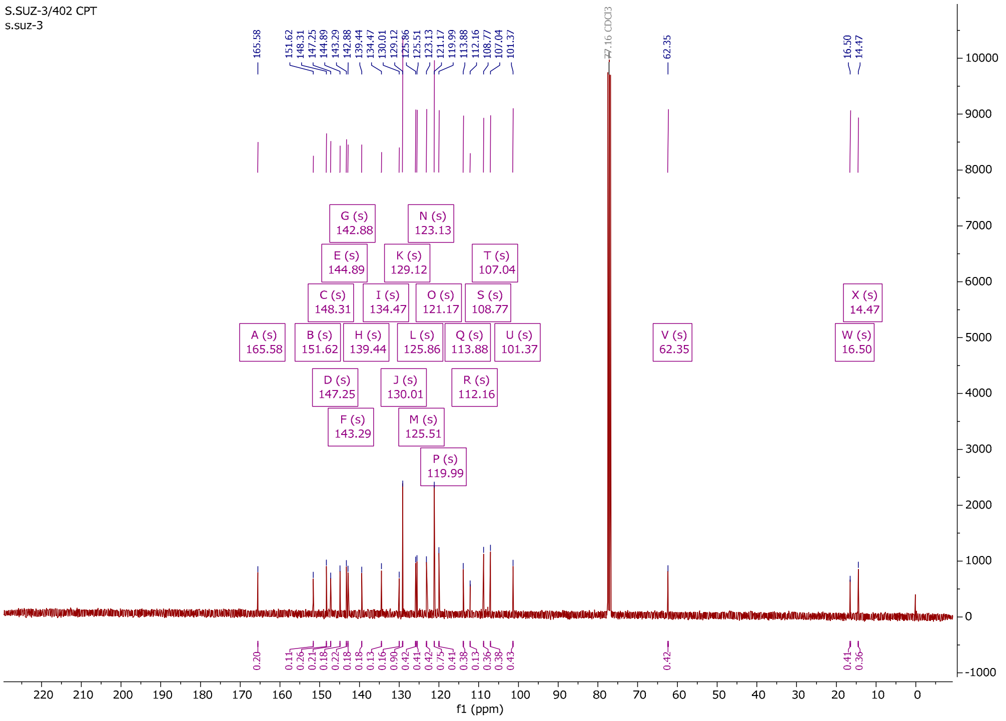


**Figure S15.** $^{13}$C-NMR spectrum of 6c.

**Ethyl 4-(5-(3,5-dimethoxyphenyl)thiophene-2-yl)-3-methyl-1-phenyl-1*H*-pyrazolo[3,4-*b*]pyridine-6-carboxylate (6d)**

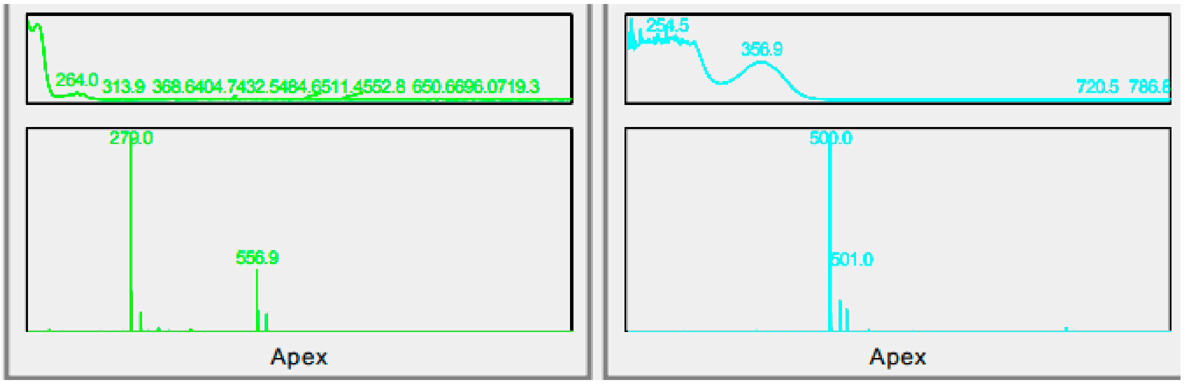


**Figure S16.** Mass spectrum of 6d.

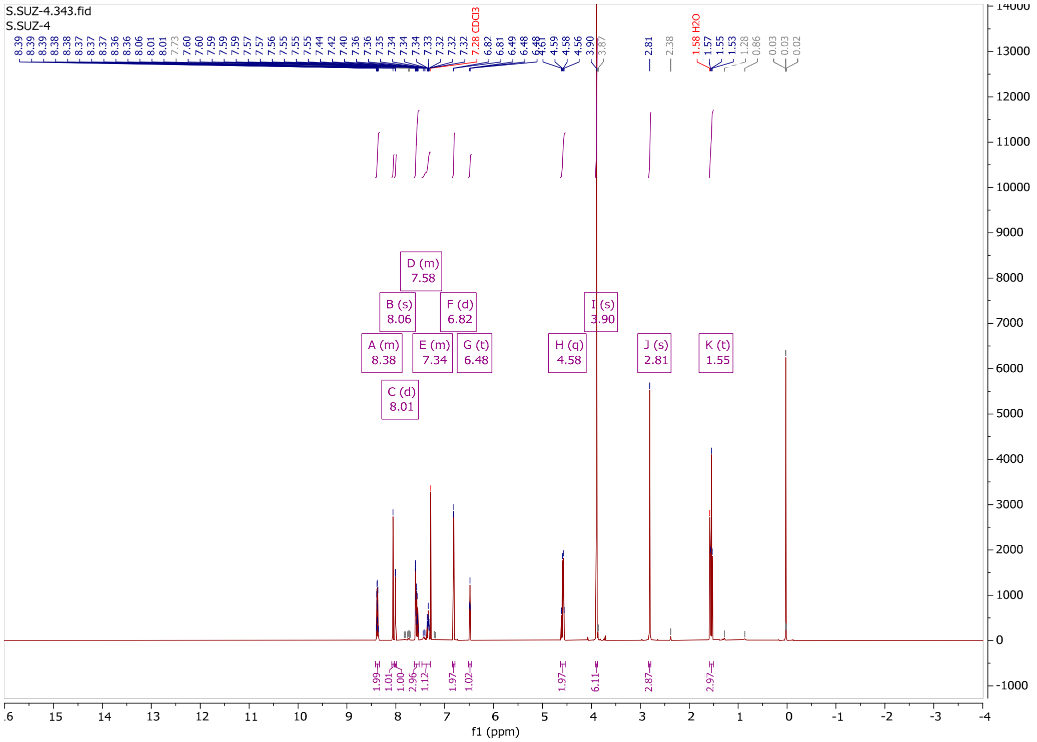

**Figure S17.** $^{1}$H-NMR spectrum of 6d.

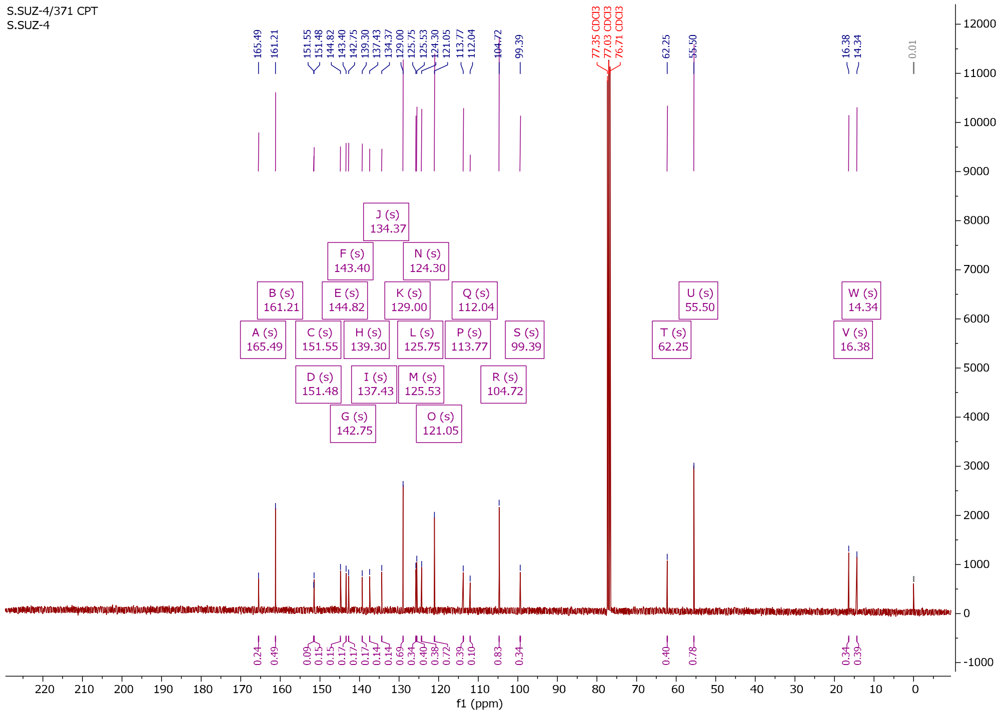

**Figure S18.** $^{13}$C-NMR spectrum of 6d.

**Ethyl 4-(5-(3,5-dimethylphenyl)thiophen-2-yl)-3-methyl-1-phenyl-1*H*-pyrazolo[3,4-*b*]pyridine-6-carboxylate (6e)**

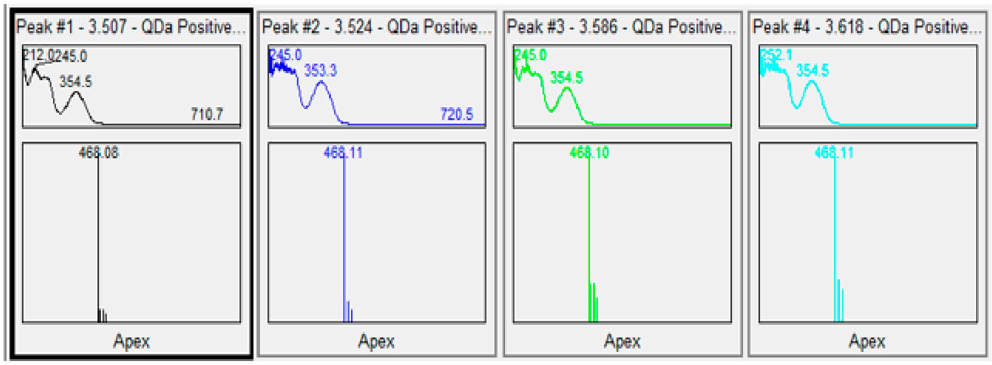


**Figure S19.** Mass spectrum of 6e.

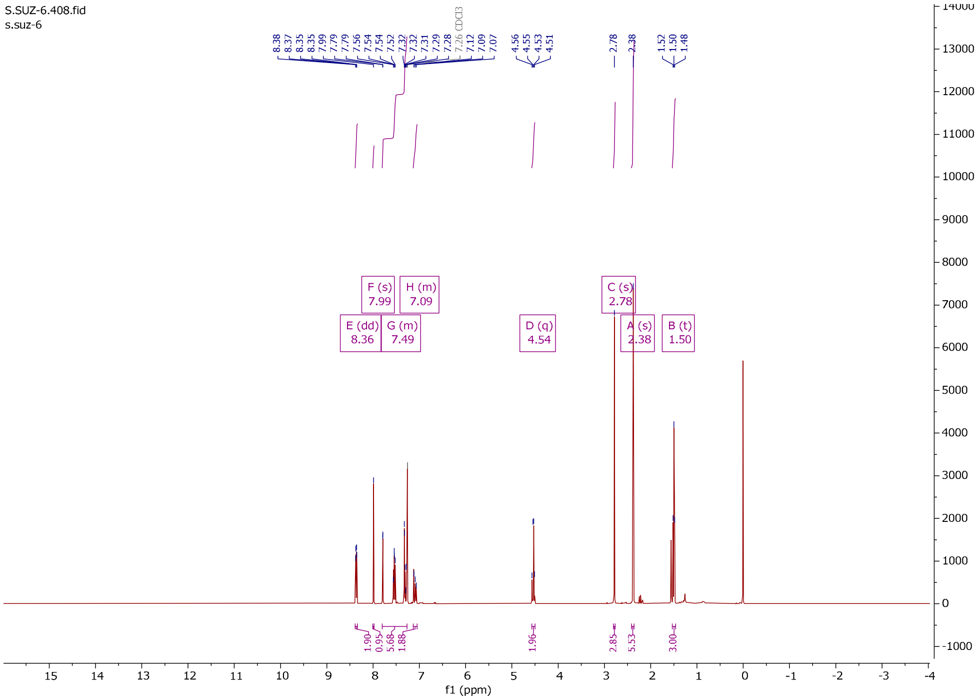


**Figure S20.** $^{1}$H-NMR spectrum of 6e.

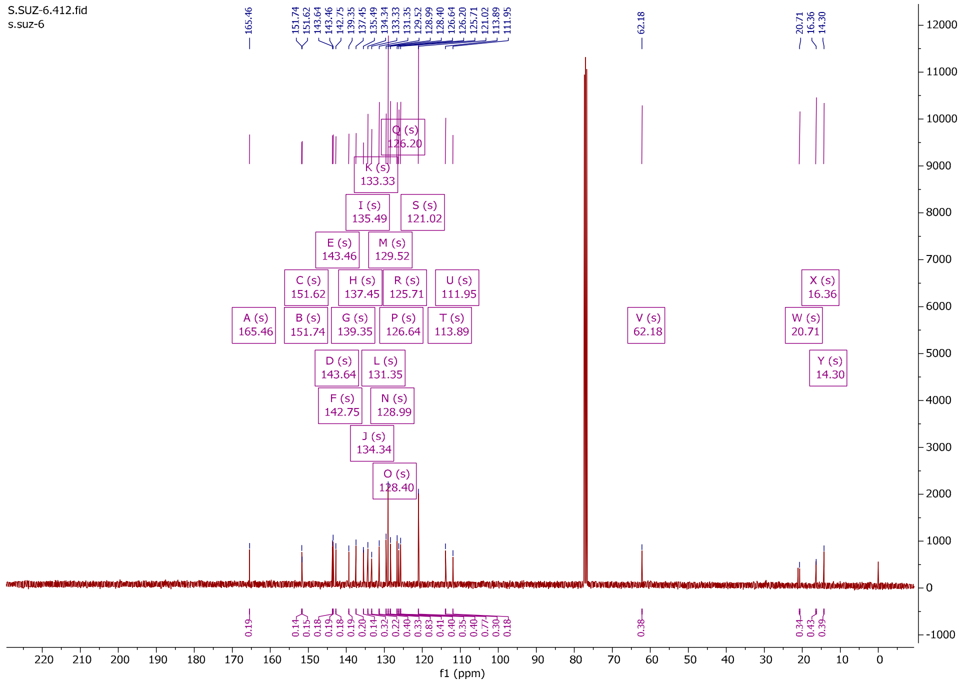

**Figure S21.** $^{13}$C-NMR spectrum of 6e.

## Ethyl 4-(5-(3-(tert-butyl)phenyl)thiophen-2-yl)-3-methyl-1-phenyl-1*H*-pyrazolo[3,4-*b*]pyridine-6-carboxylate (6f)

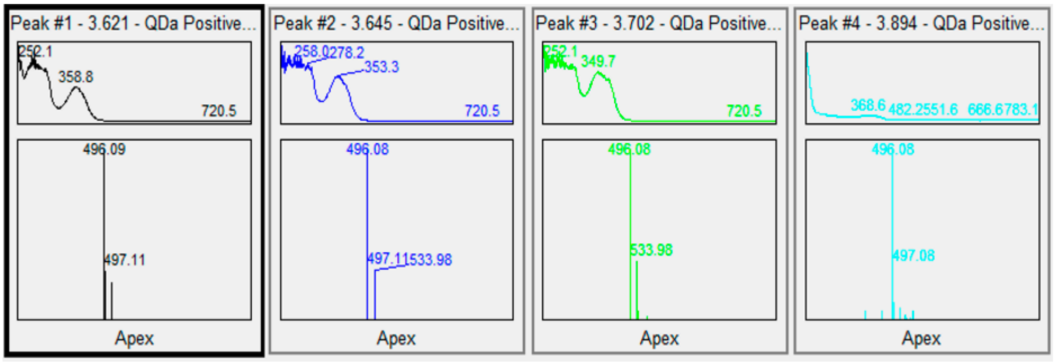


**Figure S22.** Mass spectrum of 6f.

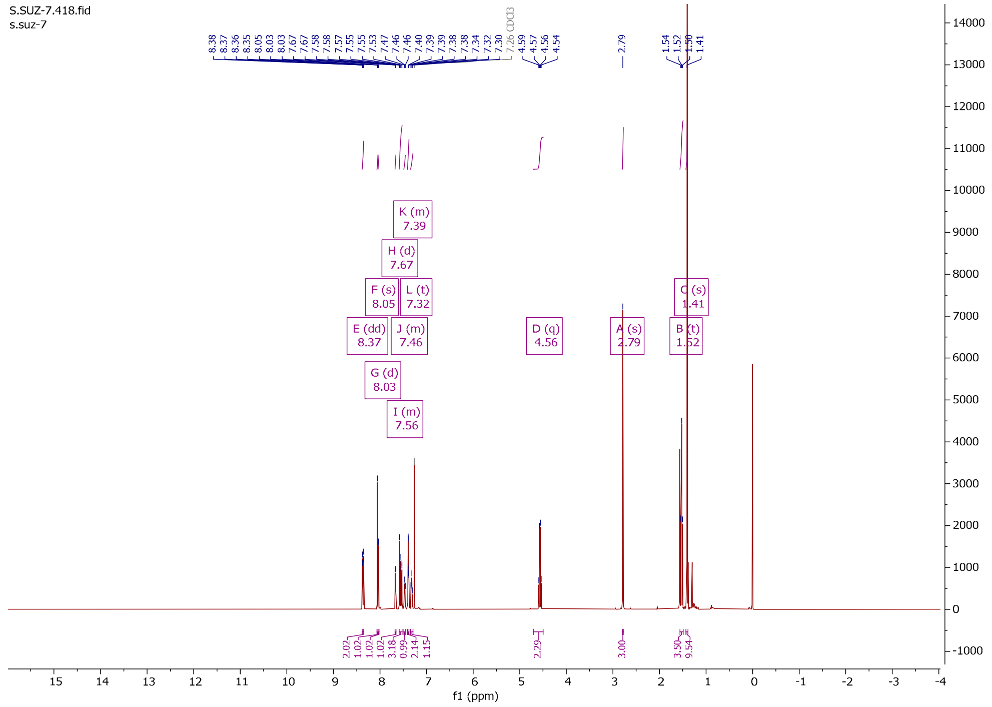


**Figure S23.** $^{1}$H-NMR spectrum of 6f.

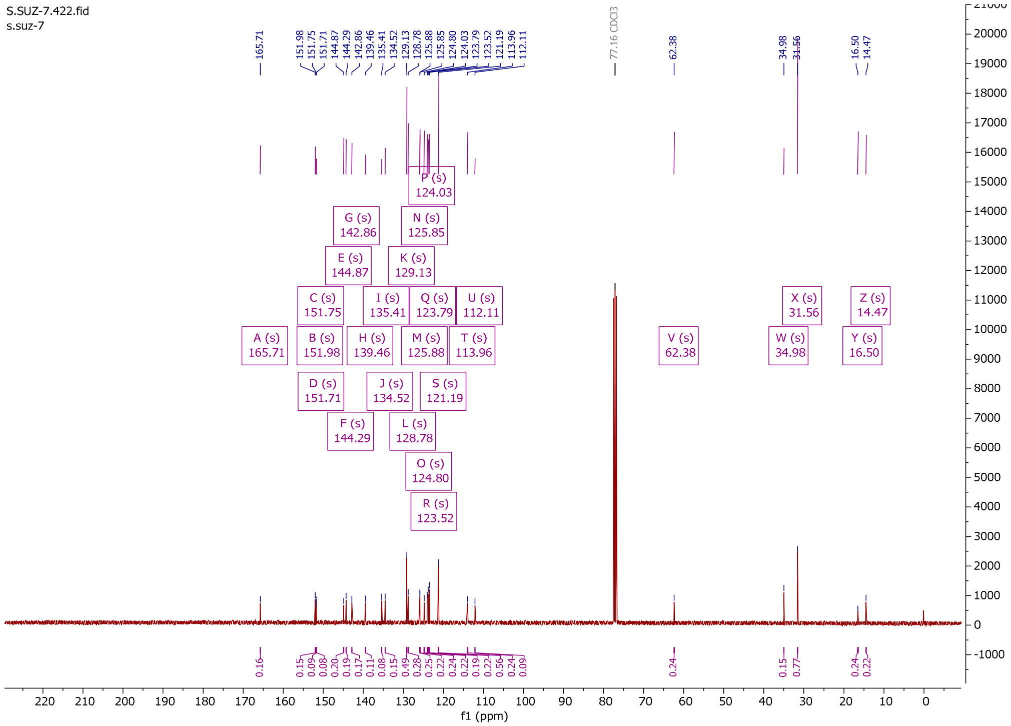


**Figure S24.** $^{13}$C-NMR spectrum of 6f.

**Ethyl 4-(5-(2,3-difluorophenyl)thiophen-2-yl)-3-methyl-1-phenyl-1*H*-pyrazolo[3,4-*b*]pyridine-6-carboxylate (6g)**

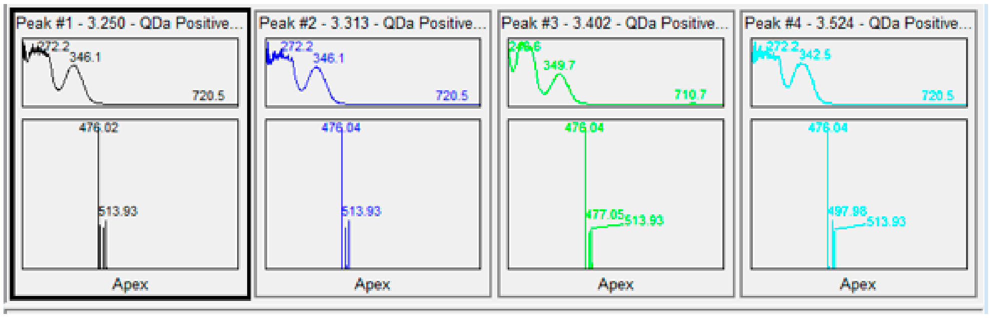


**Figure S25.** Mass spectrum of 6g.

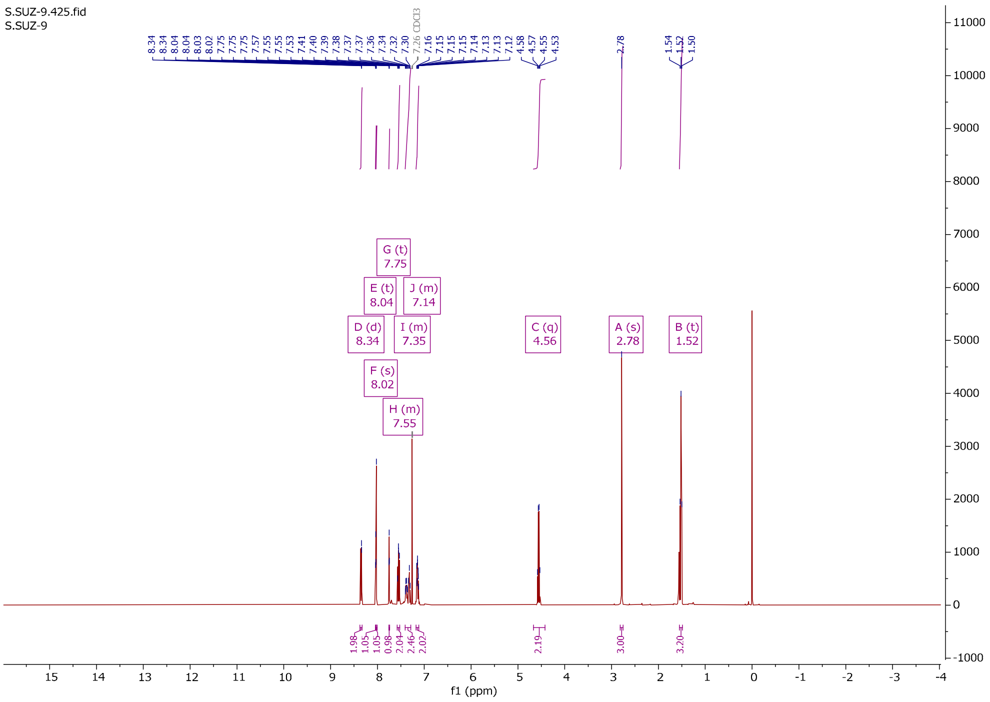


**Figure S26.** $^{1}$H-NMR spectrum of 6g.

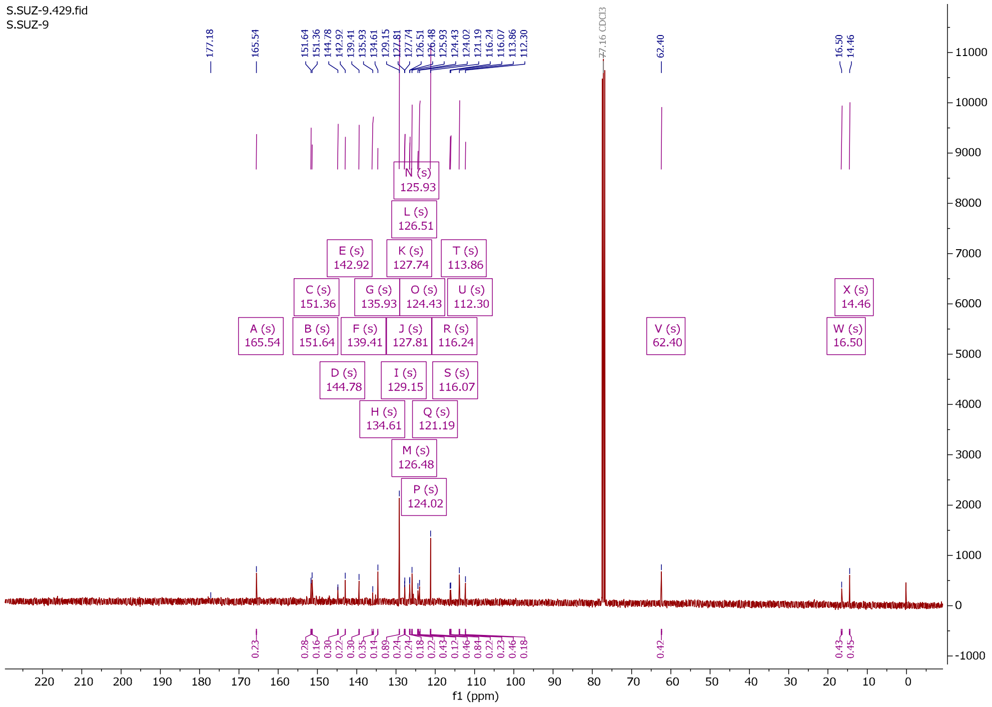


**Figure S27.** $^{13}$C-NMR spectrum of 6g.

**Ethyl 3-methyl-1-phenyl-4-(5-(3-(trifluoromethyl)phenyl)thiophen-2-yl)-1*H*-pyrazolo[3,4-*b*]pyridine-6-carboxylate (6h)**

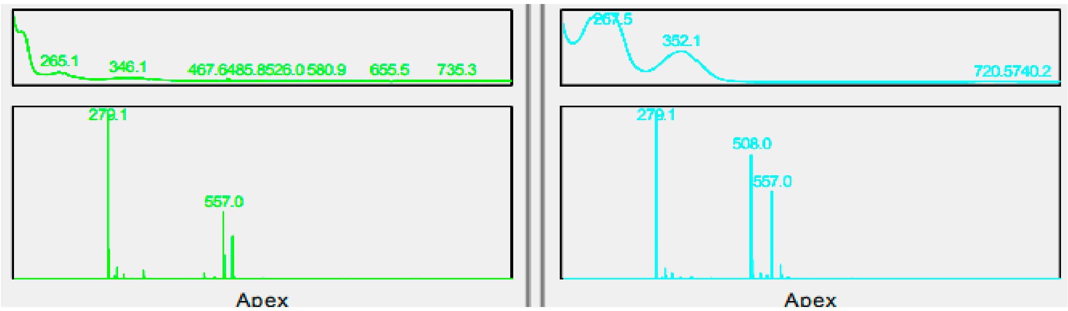


**Figure S28.** Mass spectrum of 6h.

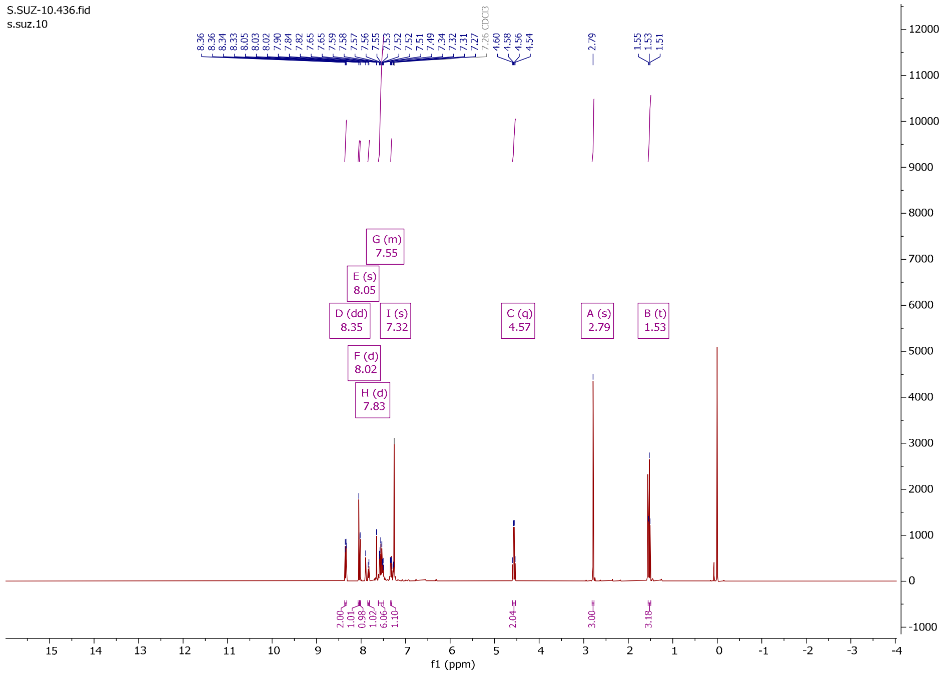


**Figure S29.** $^{1}H$-NMR spectrum of 6h.

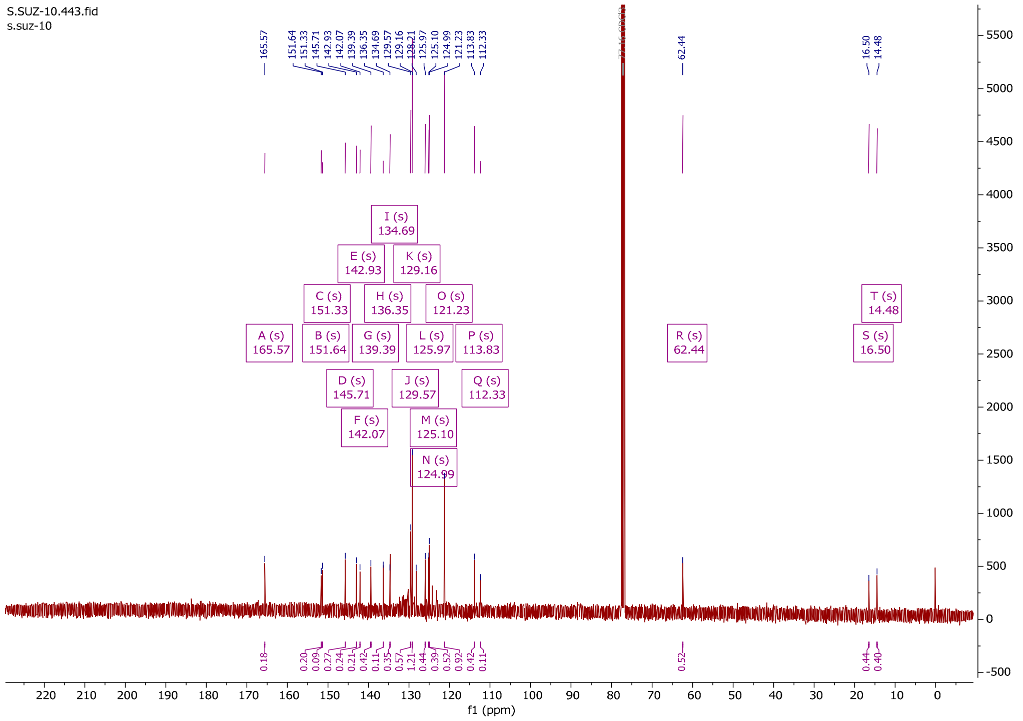


**Figure S30.** $^{13}C$-NMR spectrum of 6h.

**Ethyl 4-(5'-chloro-[2,2'-bithiophen]-5-yl)-3-methyl-1-phenyl-1*H*-pyrazolo[3,4-*b*]pyridine-6-carboxylate (6i)**

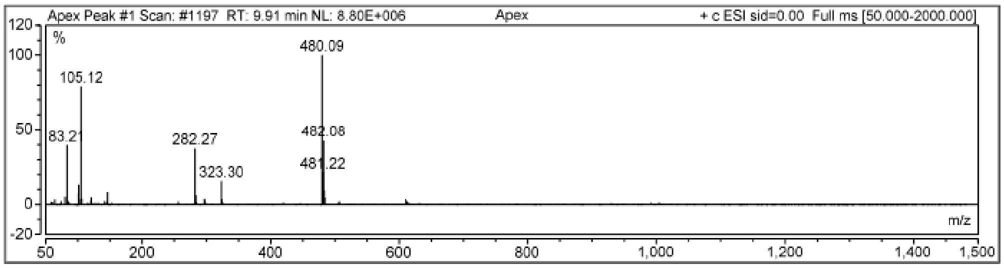


**Figure S31.** Mass spectrum of 6i.

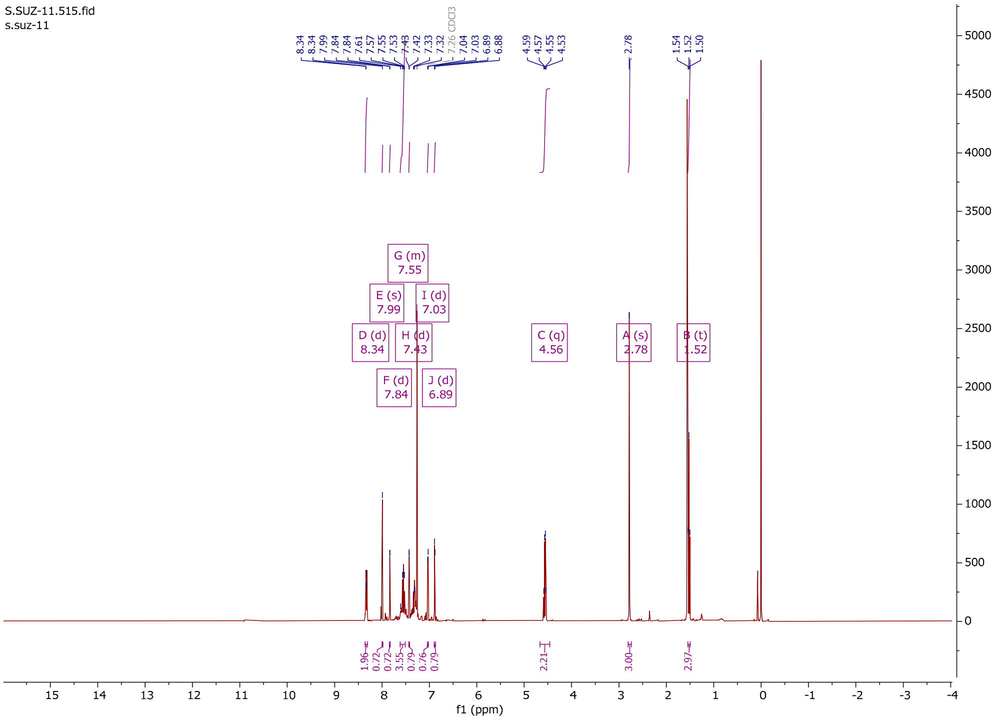


**Figure S32.** $^{1}$H-NMR spectrum of 6i.

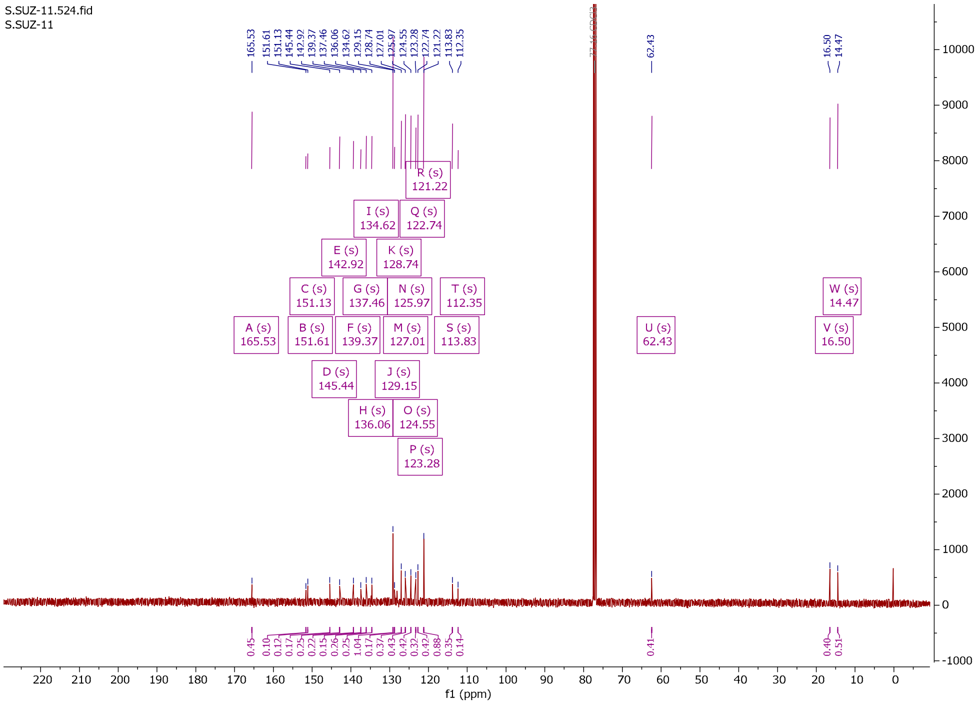

**Figure S33.** $^{13}$C-NMR spectrum of 6i.

## Ethyl-4-([2,2'-bithiophen]-5-yl)-3-methyl-1-phenyl-1*H*-pyrazolo[3,4-*b*]pyridine-6-carbohydrazide (7a)

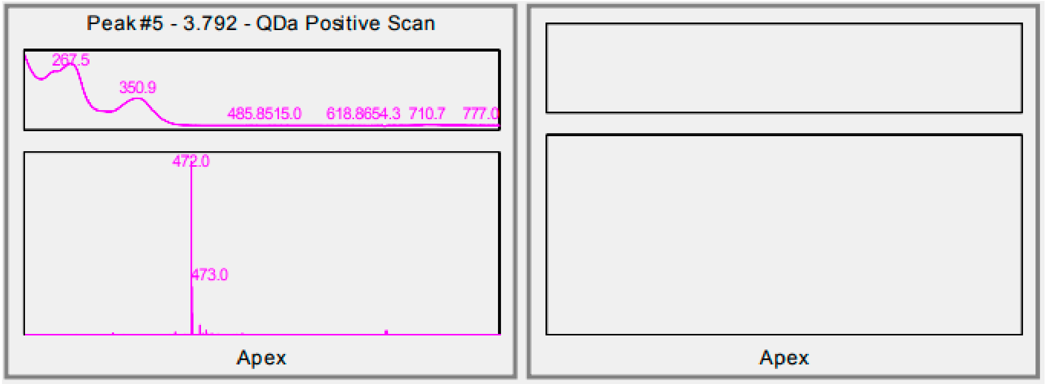


**Figure S34.** Mass spectrum of 7a.

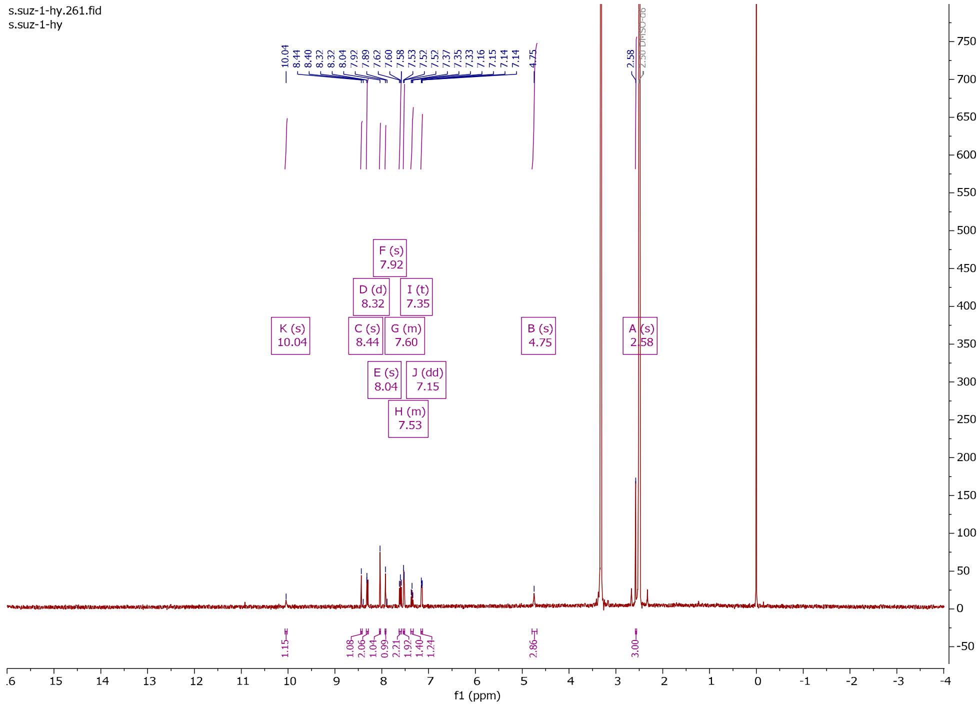


**Figure S35.** $^{1}$H-NMR spectrum of 7a.

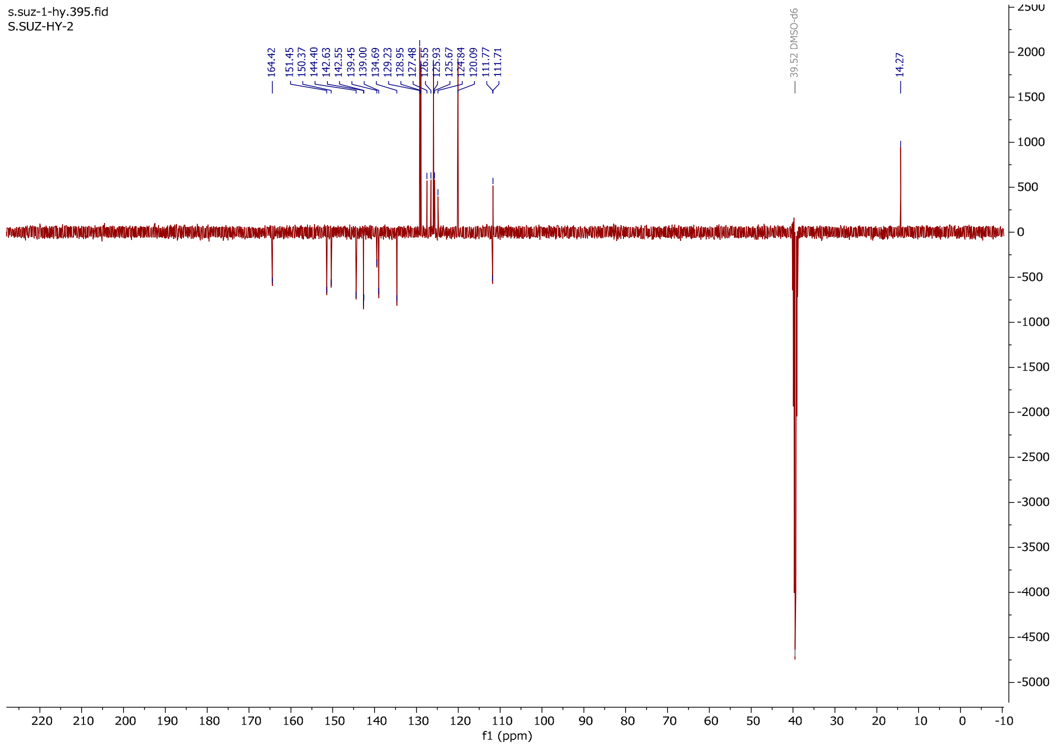


**Figure S36.** $^{13}$C-NMR spectrum of 7a.

**3-methyl-1-phenyl-4-(5-phenylthiophen-2-yl)-1*H*-pyrazolo[3,4-*b*]pyridine-6-carbohydrazide (7b)**

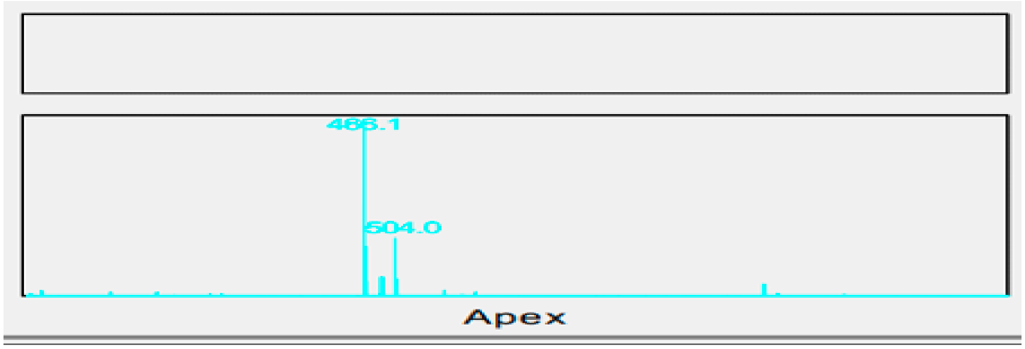


**Figure S37.** Mass spectrum of 7b.

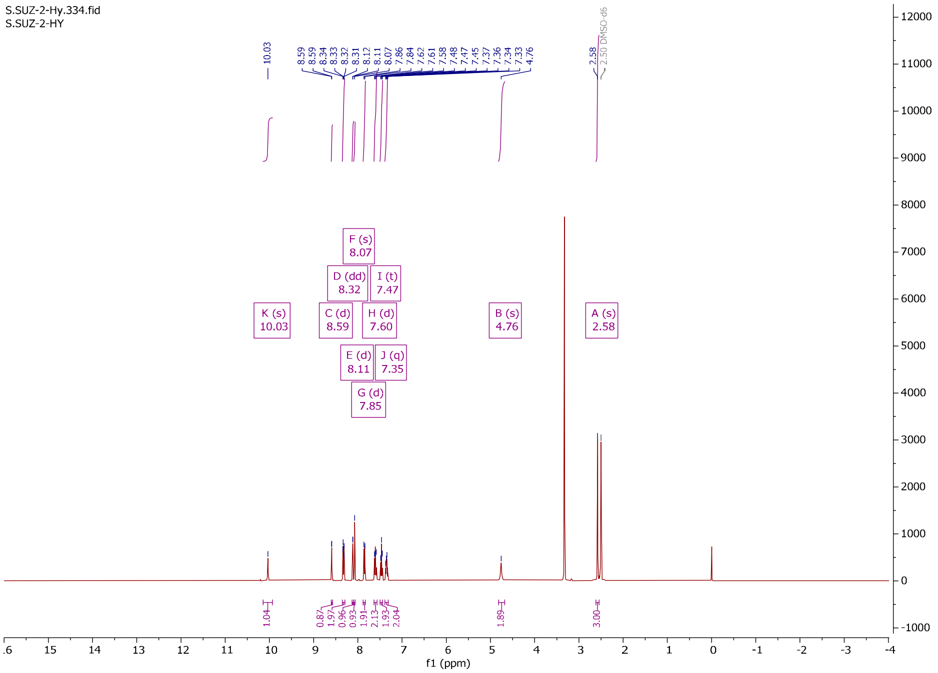


**Figure S38.** $^{1}$H-NMR spectrum of 7b.

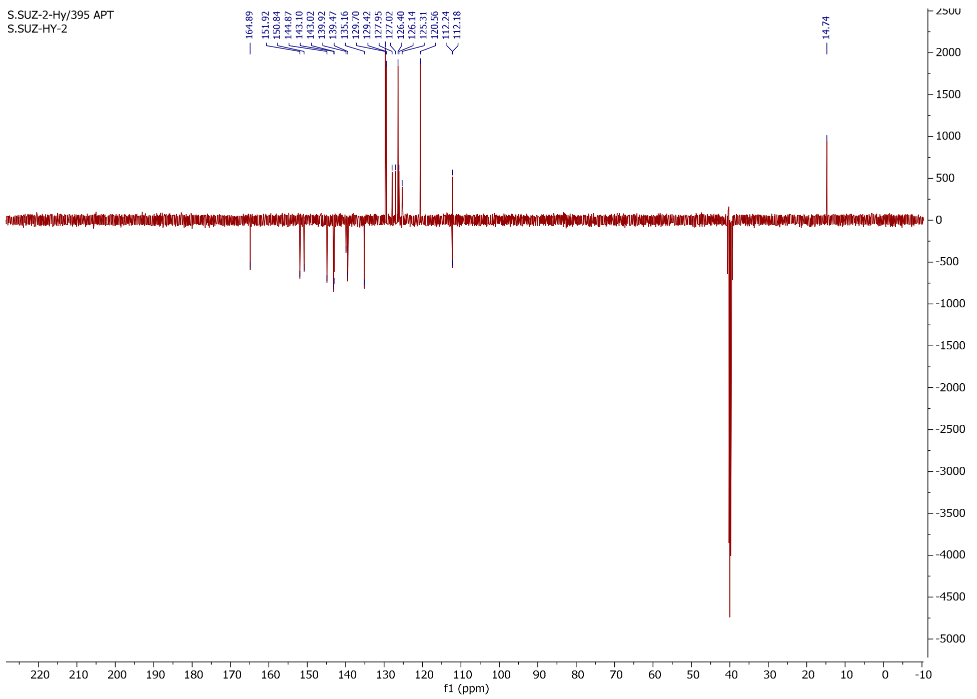


**Figure S39.** $^{13}$C-NMR spectrum of 7b.

## 4-(5-(benzo[d][1,3]dioxol-4-yl)thiophen-2-yl)-3-methyl-1-phenyl-1*H*-pyrazolo[3,4-*b*]pyridine-6-carbohydrazide (7c)

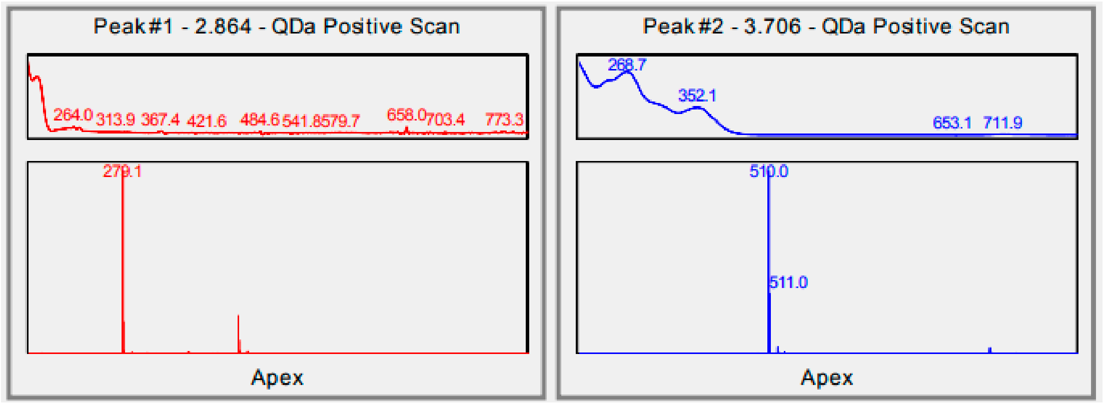


**Figure S40.** Mass spectrum of 7c.

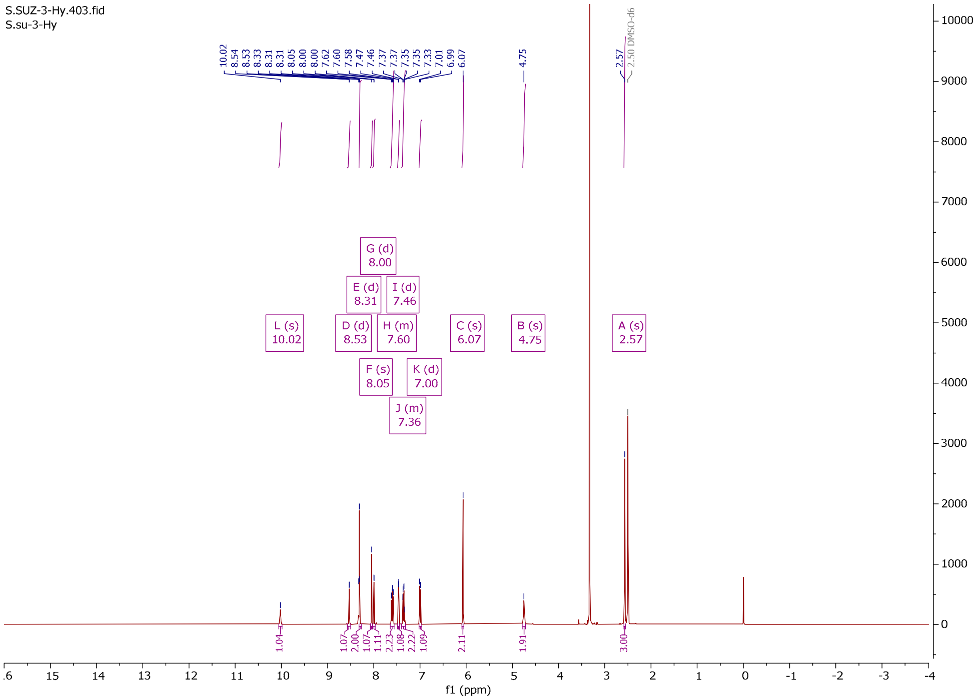

**Figure S41.** $^{1}$H-NMR spectrum of 7c.

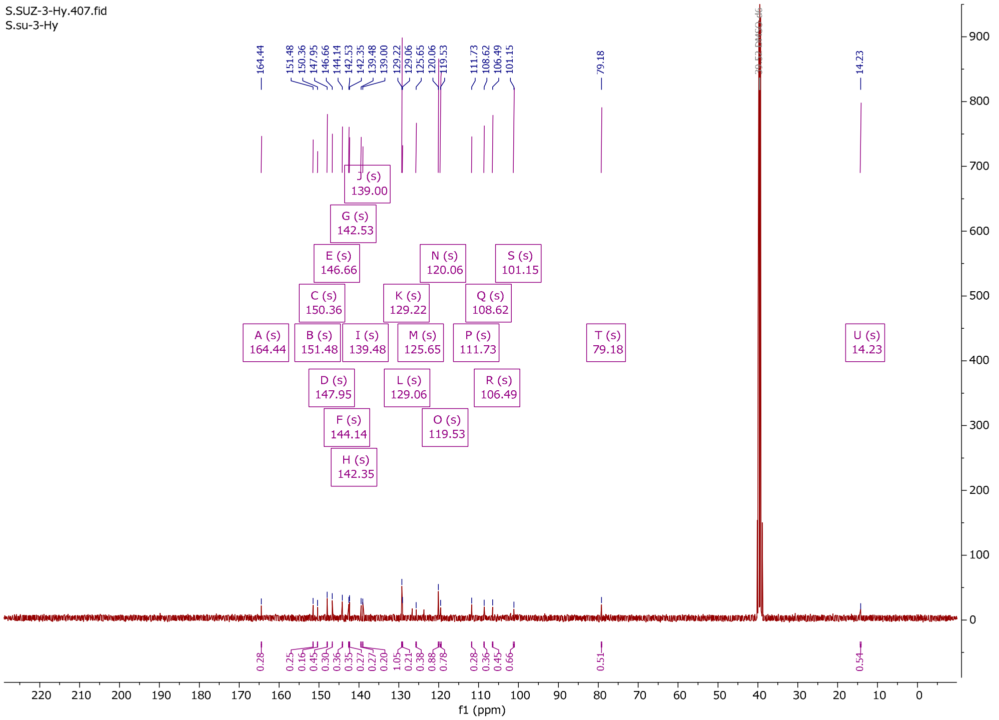

**Figure S42.** $^{13}$C-NMR spectrum of 7c.

**4-(5-(3,5-dimethoxyphenyl)thiophen-2-yl)-3-methyl-1-phenyl-1*H*-pyrazolo[3,4-*b*]pyridine-6-carbohydrazid (7d)**

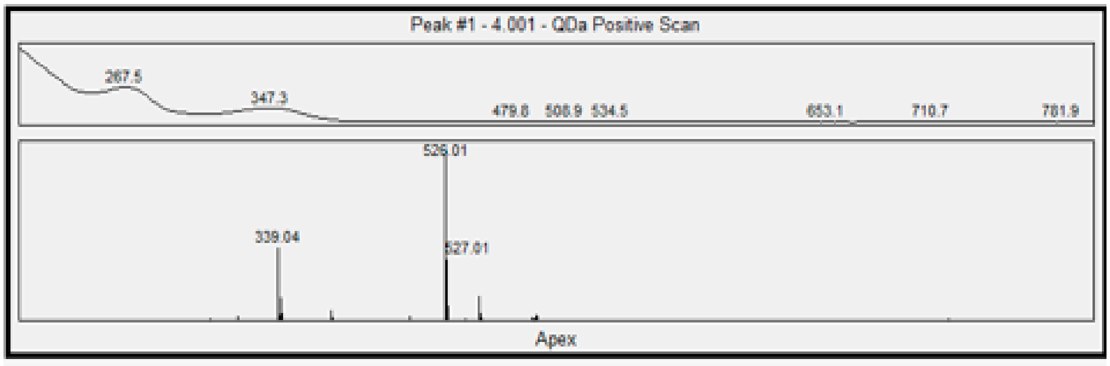


**Figure S43.** Mass spectrum of 7d.

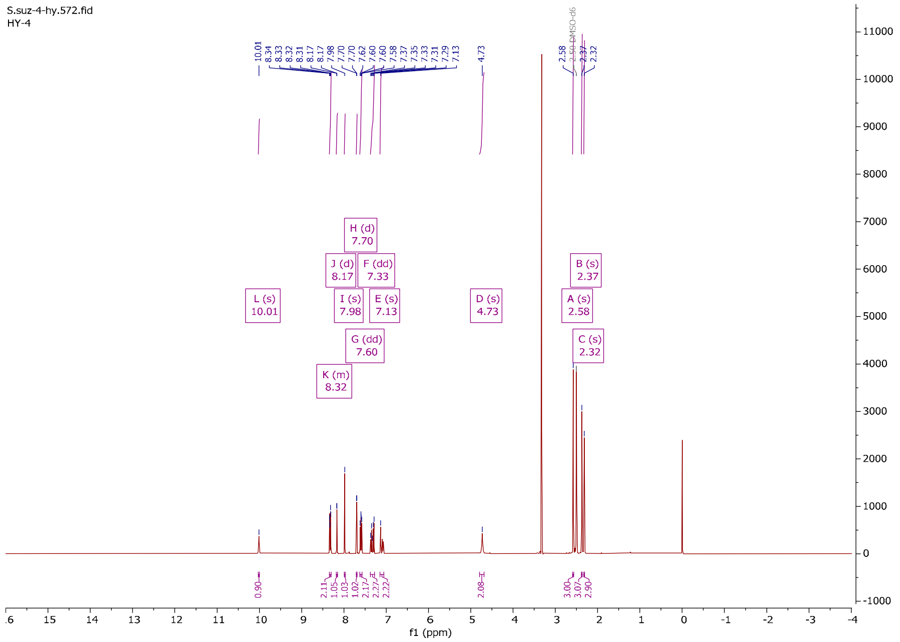


**Figure S44.** $^{1}$H-NMR spectrum of 7d.

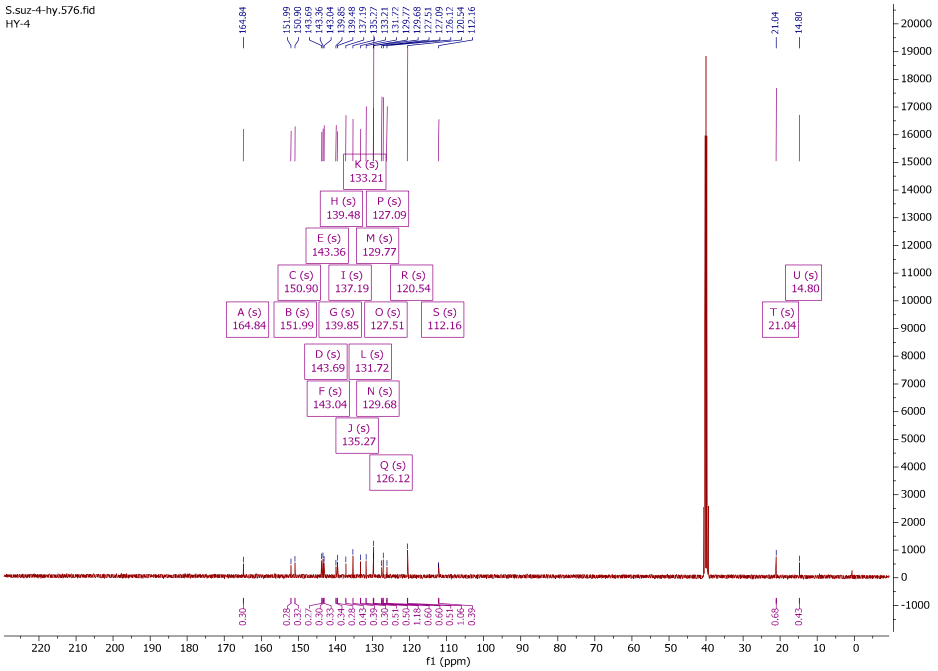


**Figure S45.** $^{13}$C-NMR spectrum of 7d.

**4-(5-(2,4-dimethylphenyl)thiophen-2-yl)-3-methyl-1-phenyl-1*H*-pyrazolo[3,4-*b*]pyridine-6-carbohydrazide (7e)**

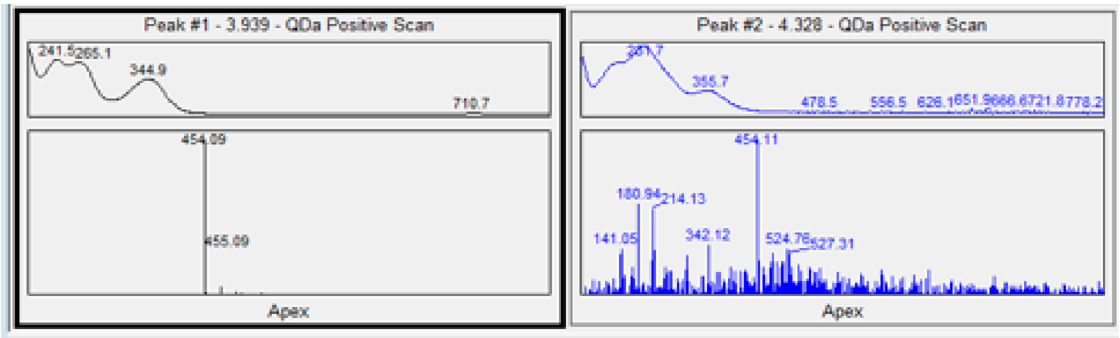


**Figure S46.** Mass spectrum of 7e.

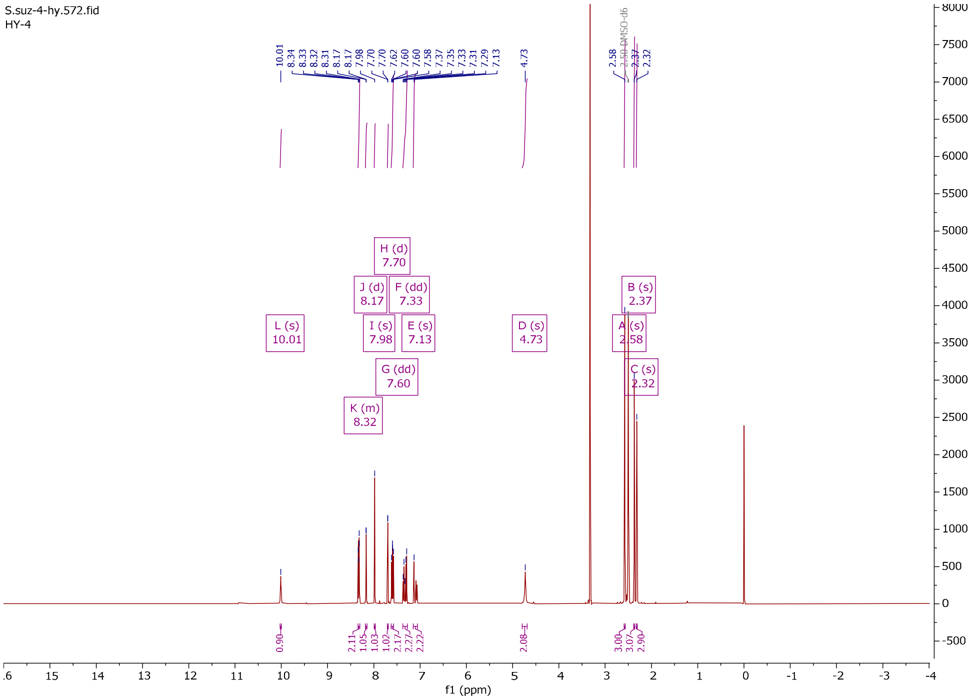


**Figure S47.** $^{1}$H-NMR spectrum of 7e.

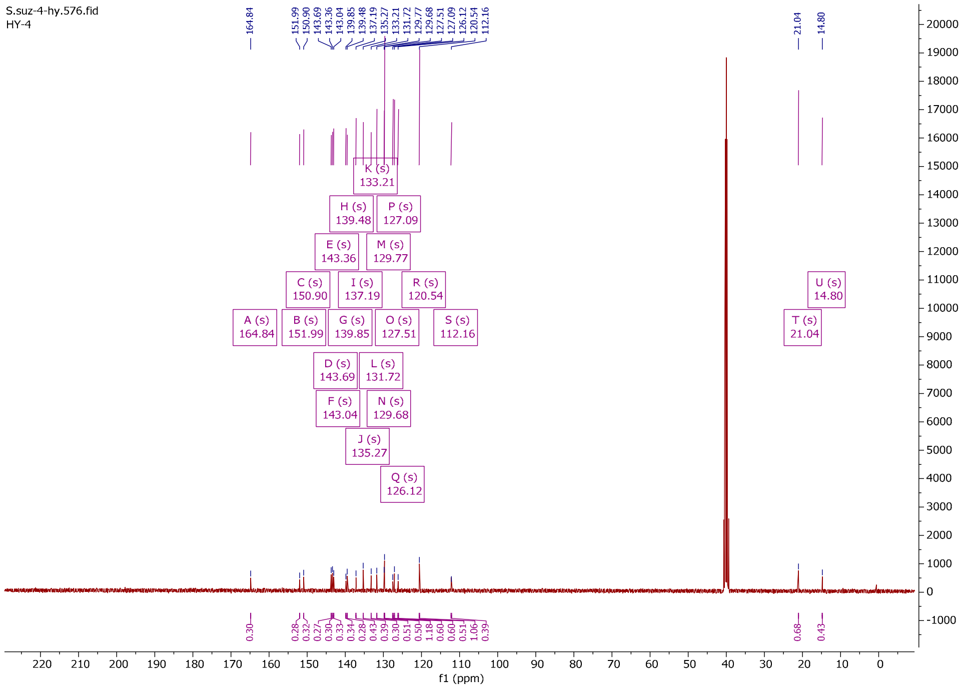


**Figure S48.** $^{13}$C-NMR spectrum of 7e.

**4-(5-(3-(tert-butyl)phenyl)thiophen-2-yl)-3-methyl-1-phenyl-1*H*-pyrazolo[3,4-*b*]pyridine-6-carbohydrazide (7f)**

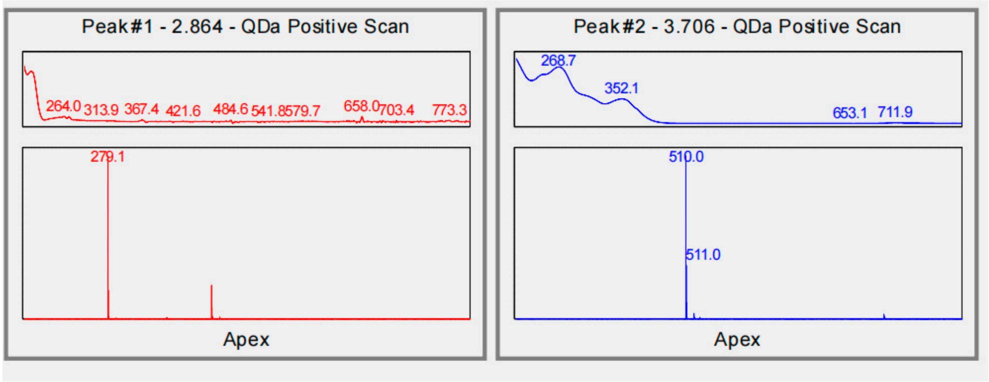


**Figure S49.** Mass spectrum of 7f.

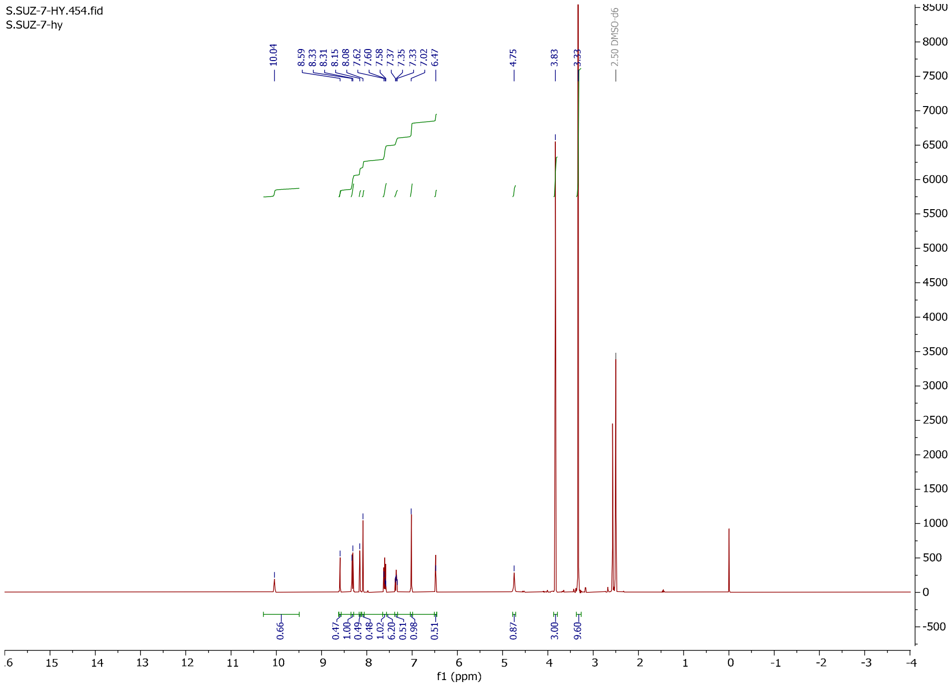


**Figure S50.** $^{1}$H-NMR spectrum of 7f.

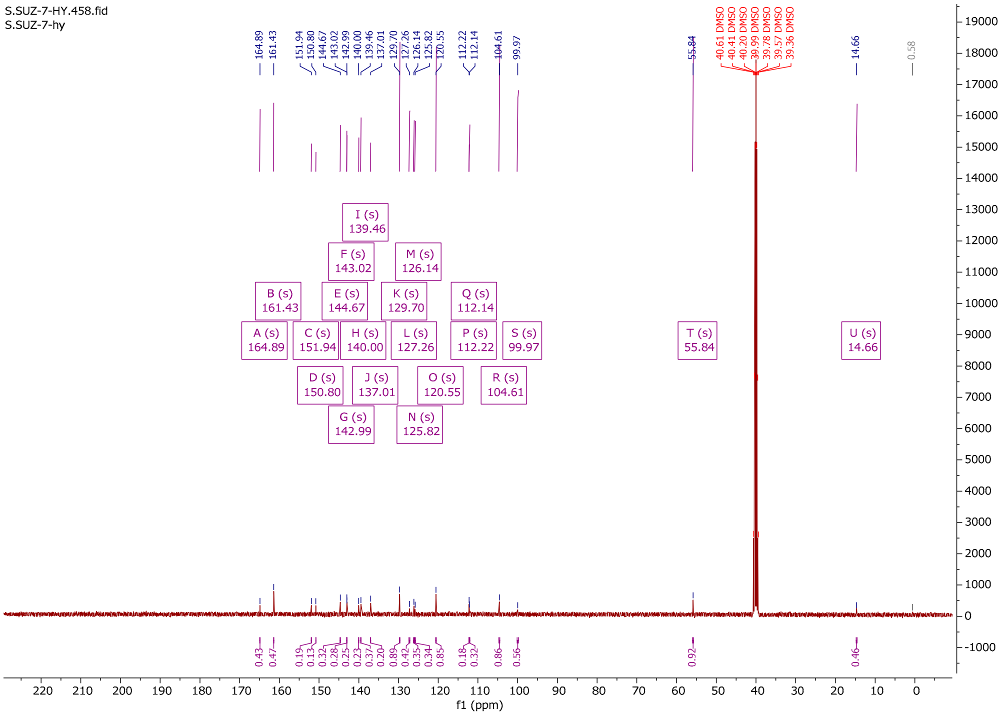


**Figure S51.** $^{13}$C-NMR spectrum of 7f.

**4-(5-(2,3-difluorophenyl)thiophen-2-yl)-3-methyl-1-phenyl-1*H*-pyrazolo[3,4-*b*]pyridine-6-carbohydrazide (7g)**

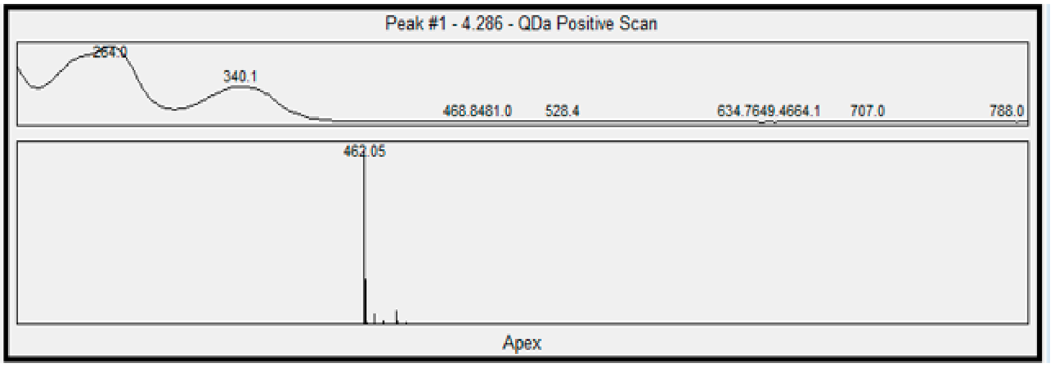


**Figure S52.** Mass spectrum of 7g.

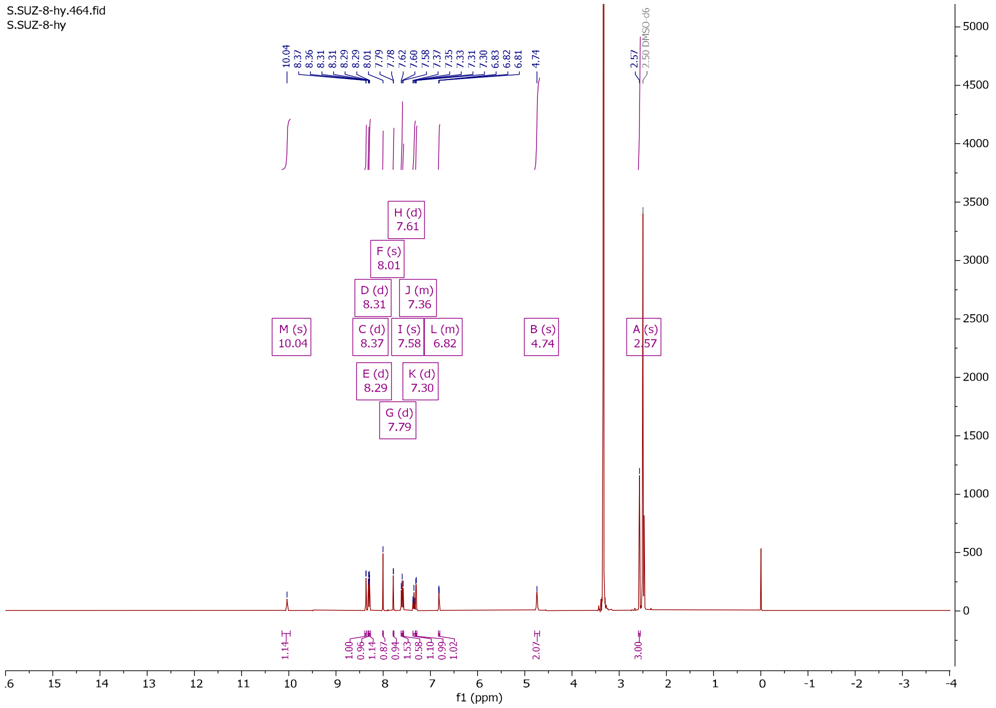

**Figure S53.** $^{1}$H-NMR spectrum of 7g.

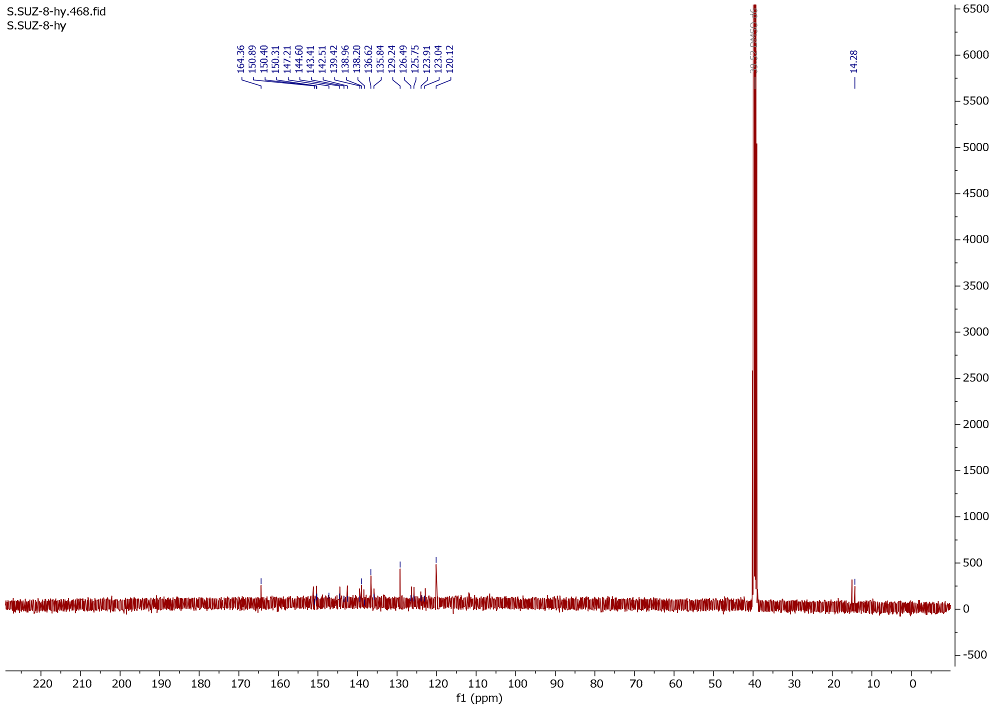

**Figure S54.** $^{13}$C-NMR spectrum of 7g.

**3-methyl-1-phenyl-4-(5-(3-(trifluoromethyl)phenyl)thiophen-2-yl)-1*H*-pyrazolo[3,4-*b*]pyridine-6-carbohydrazide (7h)**

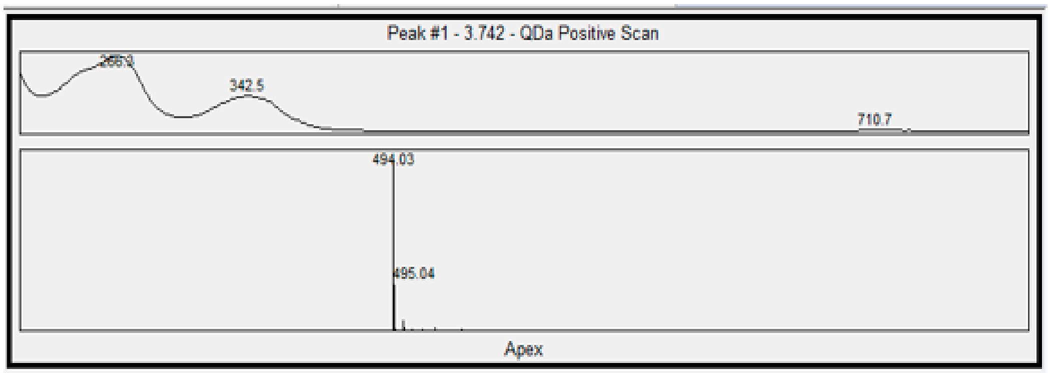


**Figure S55.** Mass spectrum of 7h.

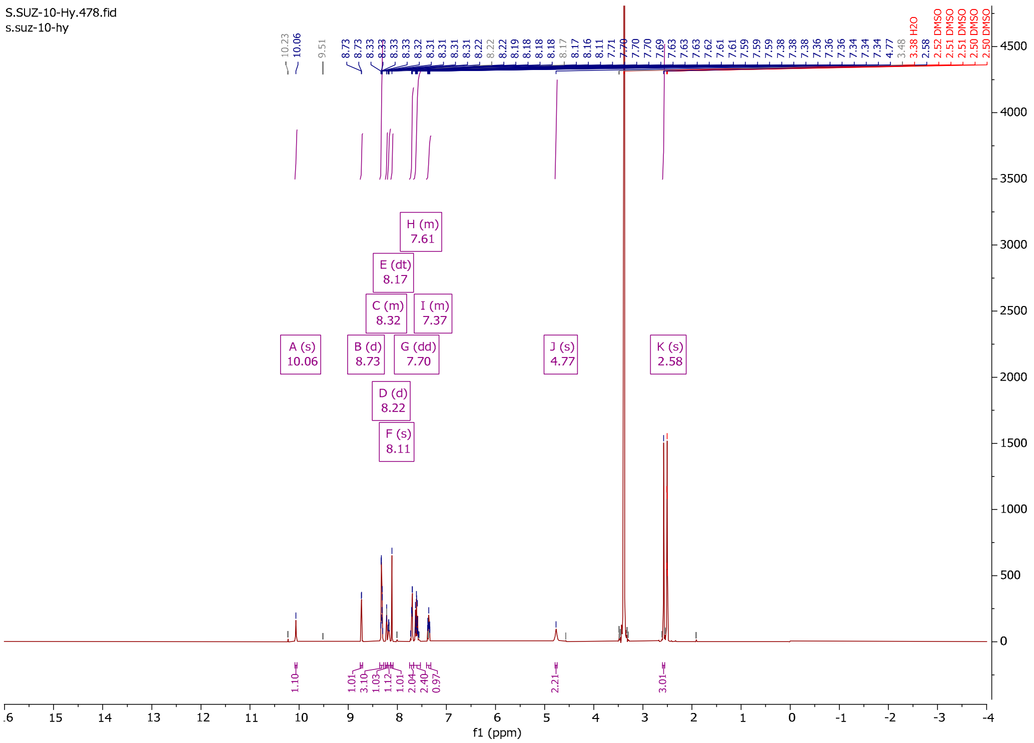


**Figure S56.** $^{1}$H-NMR spectrum of 7h.

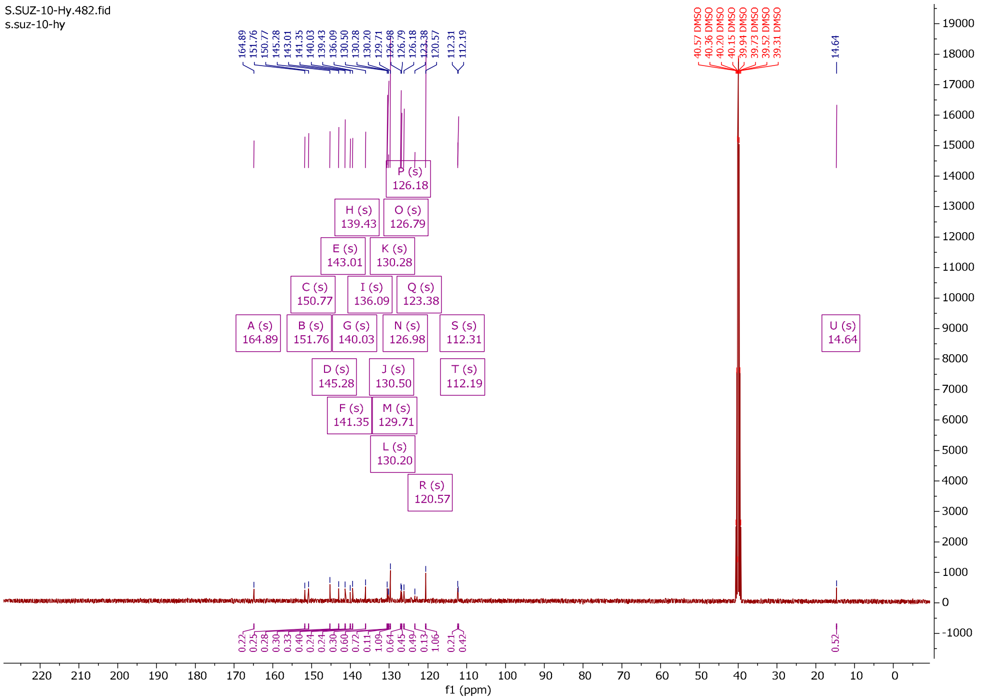


**Figure S57.** $^{13}$C-NMR spectrum of 7h.

**4-(5'-chloro-[2,2'-bithiophen]-5-yl)-3-methyl-1-phenyl-1*H*-pyrazolo[3,4-*b*]pyridine-6-carbohydrazide (7i)**

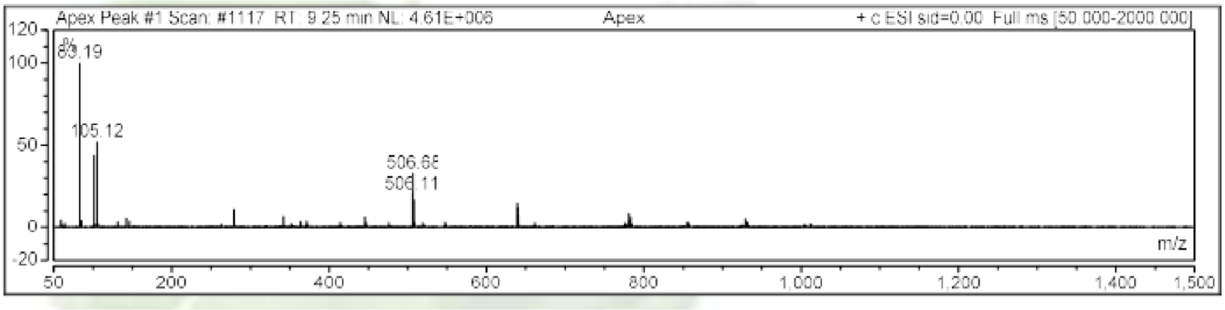


**Figure S58.** Mass spectrum of 7i.

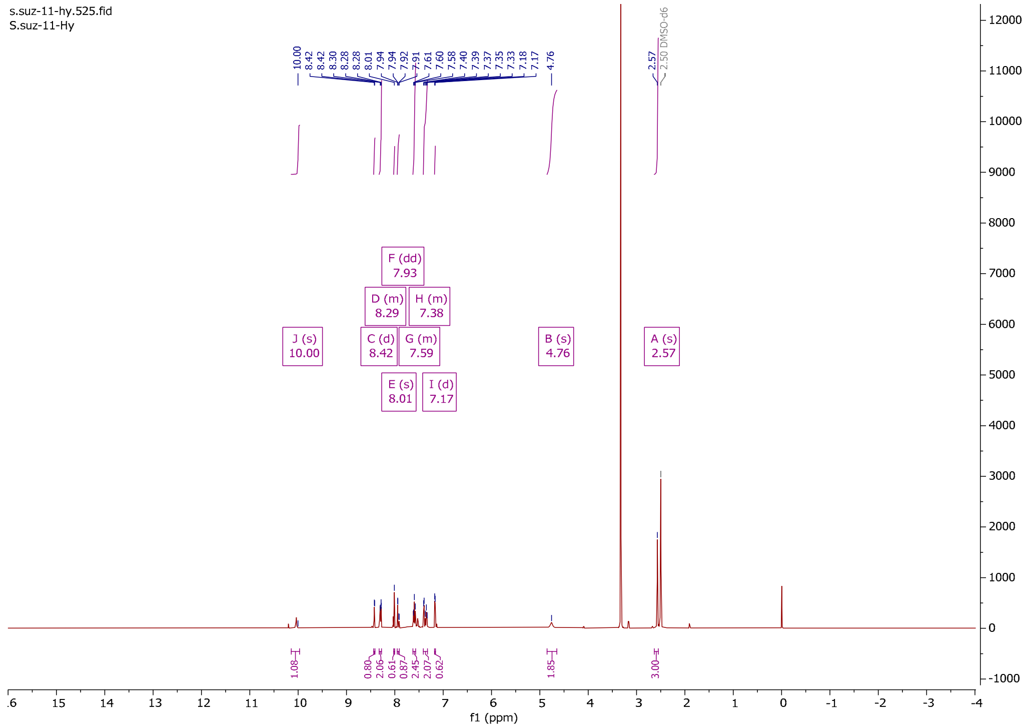


**Figure S59.** $^{1}$H-NMR spectrum of 7i.

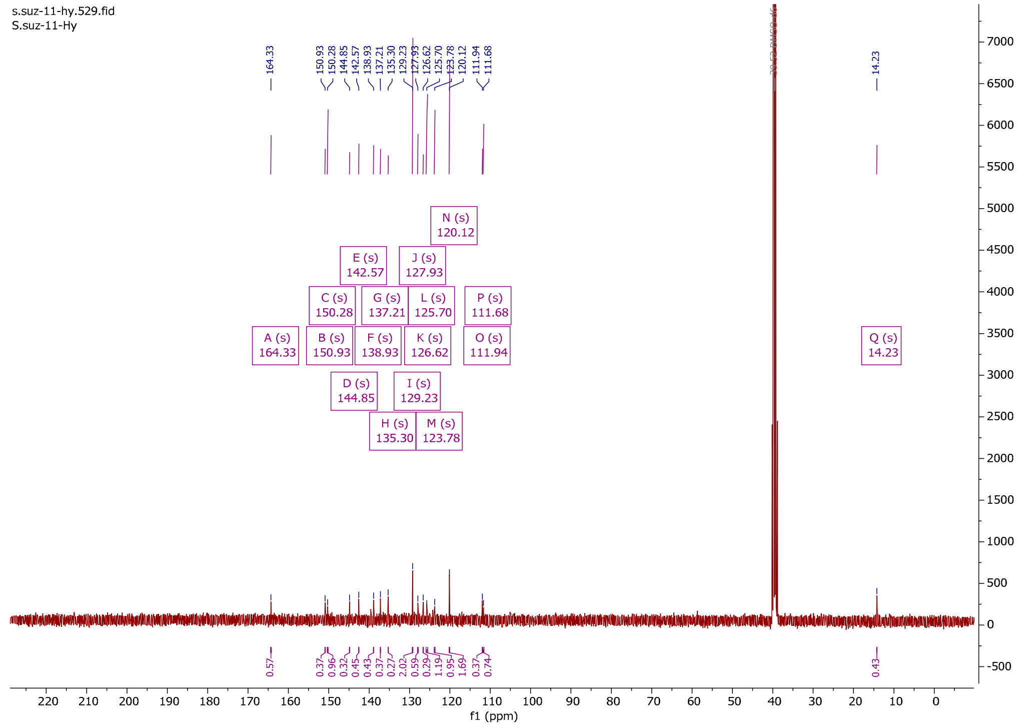


**Figure S60.** $^{13}$C-NMR spectrum of 7i.
